# CAVaLRi: An Algorithm for Rapid Identification of Diagnostic Germline Variation

**DOI:** 10.1155/2024/6411444

**Published:** 2024-04-29

**Authors:** Robert J. Schuetz, Austin A. Antoniou, Grant E. Lammi, David M. Gordon, Harkness C. Kuck, Bimal P. Chaudhari, Peter White

**Affiliations:** ^1^The Office of Data Sciences, The Abigail Wexner Research Institute, Nationwide Children's Hospital, Columbus, Ohio, USA; ^2^The Steve and Cindy Rasmussen Institute for Genomic Medicine, The Abigail Wexner Research Institute, Nationwide Children's Hospital, Columbus, Ohio, USA; ^3^Department of Pediatrics, The Ohio State University College of Medicine, Columbus, Ohio, USA; ^4^Divisions of Neonatology, Genetics and Genomic Medicine, Nationwide Children's Hospital, Columbus, OH, USA; ^5^Center for Clinical and Translational Science, The Ohio State University and Nationwide Children's Hospital, Columbus, OH, USA

## Abstract

Clinical exome and genome sequencing (ES/GS) have become indispensable diagnostic tools for rare genetic diseases (RGD). However, the interpretation of ES/GS presents a substantial operational challenge in clinical settings. Test interpretation requires the review of hundreds of genetic variants, a task that has become increasingly challenging given the rising use of ES/GS. In response, we present Clinical Assessment of Variants by Likelihood Ratios (CAVaLRi), which employ a modified likelihood ratio (LR) framework to assign diagnostic probabilities to candidate germline disease genes. CAVaLRi models aspects of the clinical variant assessment process, taking into consideration the predicted impact of the variant, the proband and parental genotypes, and the proband's clinical characteristics. It also factors in computational phenotype noise and weighs the relative significance of genotype, phenotype, and variant segregation information. We trained and tested CAVaLRi on variant and phenotype data from an internal cohort of 655 clinical ES cases. For validation, CAVaLRi's performance was benchmarked against four leading gene prioritization algorithms (Exomiser's hiPHIVE and PhenIX prioritizers, LIRICAL, and XRare) using a distinct cohort of 12,832 ES cases. Our findings reveal that CAVaLRi significantly outperforms its counterparts when clinician-curated phenotype sets are used, as evidenced by its superior precision-recall curve (PR AUC: 0.701) and average diagnostic gene rank (1.59). Notably, even when substituting highly focused clinician-curated phenotype sets with large and potentially nonspecific computationally derived phenotypes, CAVaLRi retains its precision (PR AUC: 0.658; diagnostic gene average rank: 1.68) and markedly outperforms other tools. In a large, heterogeneous validation cohort, CAVaLRi stood out as the most precise prioritization algorithm (PR AUC: 0.335; average diagnostic rank: 1.91). In conclusion, CAVaLRi presents a robust solution for prioritizing diagnostic genes, surpassing current methods. It demonstrates resilience to noisy, computationally-derived phenotypes, providing a scalable strategy to help labs focus on the most diagnostically relevant variants, thus addressing the growing demand for ES/GS interpretation.

## 1. Introduction

Rare genetic diseases (RGDs) affect 8% of the U.S. population and collectively represent the leading cause of infant mortality [1]. Multiple studies demonstrate the importance of an accurate diagnosis in cases of suspected RGD, including a more precise prognosis, increased cost-effectiveness of care, and risk assessment in future family planning [2–4]. While exome and genome sequencing (ES/GS) are increasingly relied upon for diagnosing RGD, they are not without challenges. Compared to a reference genome, patients are expected to have 4-5 million genetic variants, primarily single nucleotide variants (SNVs), but also insertions, deletions (indels), and structural variants. After removing noncoding variants (i.e., focusing on the ~2% of the human genome that is exonic), common variants, and those annotated as having benign significance, hundreds of potentially disease-causing candidate variants remain for manual review [5, 6]. Given the scale of the problem, efficient prioritization of potential disease-causing variants is a critical need [7].

For each candidate variant identified in ES/GS, clinical genomicists assess the pathogenicity of the variant (either implicitly or explicitly by applying a variant assessment framework [8]) and the concordance of the patient's phenotypes with phenotypic frequencies in disease(s) associated with the gene of interest [9]. Efforts to curate gene-disease associations have led to the creation of databases, such as the Human Gene Mutation Database [10] and Online Mendelian Inheritance in Man (OMIM) [11], that aim to catalog the thousands of known RGDs and their molecular bases. Similarly, disease-phenotype annotations have been standardized under the Human Phenotype Ontology (HPO) [12]. In addition to database comparison, clinical genomicists analyze available parental ES/GS data to ascertain whether candidate variants segregate in a manner consistent with the associated diseases' mode of inheritance (MOI).

Despite the availability of these valuable resources, the limited pool of experts capable of performing variant assessment, compounded by the sheer number of candidate variants they must assess per case [13–15], precludes current processes from scaling to meet the growing demand for ES/GS testing [3]. In addition to the initial ES/GS analysis, reanalysis of previously unsolved ES/GS, powered by emerging gene-disease associations or the phenotypic evolution of individual patients, is an increasingly recognized means of diagnosing RGDs [16]. Given that the diagnostic yield of initial ES/GS is 25-45% [17–21], as the initial use of ES/GS grows, the number of unsolved ES/GS cases from years prior also grows. Considering these factors, the need for scalable and reproducible variant prioritization in ES/GS analysis has never been greater.

Several computational approaches have been proposed that model the human-directed, manual process of reviewing a genetic variant for diagnostic potential [22]. When comparing variant prioritization algorithms to human-directed efforts in our hospital's clinical molecular genetics laboratory, it became evident that computational approaches do not always consider parental genotype and commonly fail to account for the relative importance between genotype and phenotype. We developed the Clinical Assessment of Variants by Likelihood Ratios (CAVaLRi) framework to more accurately prioritize potentially diagnostic genetic variation. Additionally, CAVaLRi is designed to handle large, noisy, computational phenotypes and thus can be combined with natural language processing (NLP) algorithms to automate diagnostic variant prioritization (a feature particularly beneficial in reinterpretation). By increasing the accuracy of diagnostic variant (and by extension, gene and disease) prioritization, the assessment burden per patient decreases, enabling scalability in an environment of growing demand for ES/GS.

## 2. Materials and Methods

### 2.1. Data Used for Model Training, Testing, and Validation

#### 2.1.1. Training and Testing Cohort: Local Suspected RGD Clinical ES Cohort

Phenotype and ES data were curated from 655 patients suspected of RGD (clinical ES), 203 of whom were determined to have one or more diagnostic variant(s) recorded in their clinical record. This data was generated from Nationwide Children's Hospital (NCH) patients evaluated at The Steve and Cindy Rasmussen Institute for Genomic Medicine (IGM) Clinical Laboratory. This molecular genetics laboratory is fully accredited by the College of American Pathologists and certified according to the Clinical Laboratory Improvement Amendments (CAP/CLIA). As part of the routine diagnostic testing process, phenotype descriptions from the medical record were manually mapped to HPO terms by laboratory genetic counselors through a phenotype curation interface developed at NCH (manual phenotypes). Computationally derived phenotype terms were extracted from prediagnostic electronic health records using ClinPhen [23] for 586 clinical ES cases where clinical notes were available (computational phenotypes). This previously published tool extracts HPO terms and has demonstrated utility in generating computed phenotypes in cases of suspected RGD [23]. The 69 clinical ES cases without available notes were excluded from computational versus manual phenotype assessment (see Algorithmic Comparison).

#### 2.1.2. External Validation Cohort: Deciphering Developmental Disorders RGD ES Cohort

**Figure 1:**
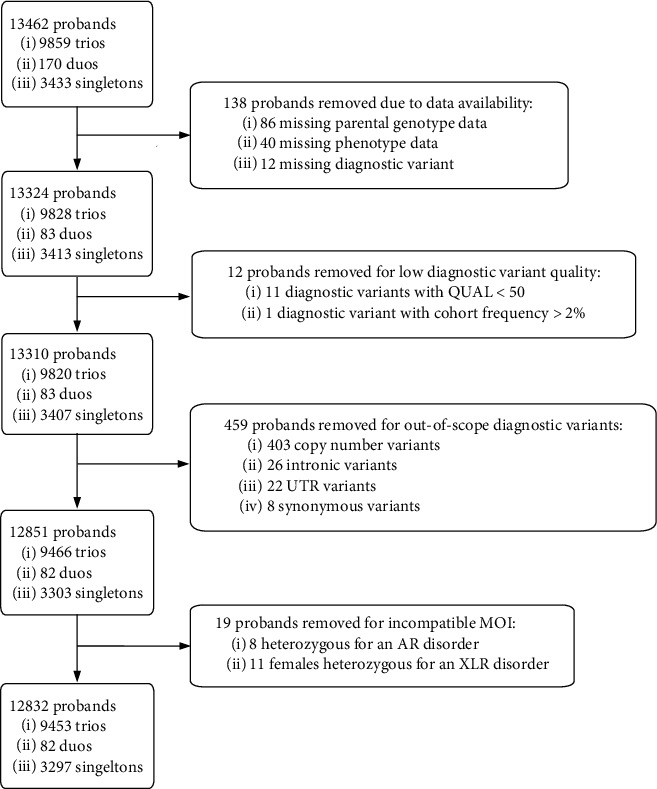
DDD CONSORT diagram. 13,642 cases from the DDD cohort were initially considered before narrowing the final case count to 12,832. 138 proband were removed due to expected data not being present (sample missing in VCF, no phenotypes listed, or diagnostic variant missing in VCF). 12 proband were removed due to low quality or high cohort frequency. 459 probands were removed due to lying outside of the scope of CAVaLRi detection (CNVs, nonexonic, and synonymous function, see Figure 3(b), Supplemental Table 1). 19 probands were removed due to disagreement between patient genotype and annotated mode of inheritance (heterozygous variants causing recessive conditions; Supplemental Table 2).

For external validation, 13,462 RGD patients from the Deciphering Developmental Disorders (DDD, EGAS00001000775) study [24] were initially considered, 12,823 of which were included in the final analysis (Figure 1). DDD provides germline variant calls for patients and their parents, as well as the patient's phenotypic abnormalities encoded in HPO terms. Of the DDD cases analyzed, 3,615 (28.2%) were considered diagnosed by exonic or splice region findings according to a recently published diagnostic variant set [25] (Table 1). This validation cohort's phenotype was treated as clinician-curated phenotypes, as DDD does not provide access to original medical records.

#### 2.1.3. Variant Calling

Clinical ES variants in the local cohort were called using Churchill [26], a best practice implementation of sequence read alignment to the GRCh38 human reference genome, base quality score recalibration, and genotyping that the IGM clinical laboratory utilizes. The resulting variant call format (VCF) files and provided DDD VCF files were independently annotated with intracohort frequencies. Following annotation, filtering was applied to exclude variants of low quality (QUAL < 50) and variants occurring at a frequency greater than 2% within respective cohorts. In the DDD germline VCFs, variants with a VSQLOD (for variant quality score log-odds) FILTER value were changed to PASS, as several diagnostic variants were assigned a VSQLOD value.

### 2.2. Clinical Assessment of Variants by Likelihood Ratios (CAVaLRi) Framework Overview

A likelihood ratio (LR) framework for OMIM disease prioritization was previously detailed in LIRICAL [27]. In a curated RGD cohort, LIRICAL demonstrated superior performance compared to Exomiser's PhenIX and hiPHIVE prioritizers [28]. This framework allows for the independent scoring of concepts that are critical in determining the clinical significance of a genetic variant, namely, the impact on functional regions (LR_geno_) and phenotypic overlap with candidate diseases (LR_pheno_). Mathematically, a product of these LRs can be used to scale the likelihood that a given variant is causing the disease phenotype. Along with a known prior probability, this composite LR can yield a posterior probability of a certain RGD being present, which is preferable for human interpretation.

**Figure 2:**
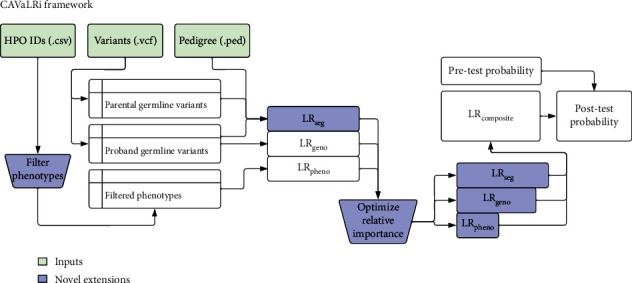
CAVaLRi implements novel extensions to the LR paradigm. LIRICAL first described a likelihood ratio model for prioritizing disease-causing genetic variants; however, some critical clinical components of the diagnostic process were not represented. Novel components incorporated into the extended framework are indicated in blue and include incrementing filtered phenotype sets, adding a mode of inheritance likelihood ratio (LR_seg_) and rescaling the relative importance of each likelihood ratio component. CAVaLRi takes a list of HPO IDs (.csv format), a list of variants (.vcf format), and a pedigree file detailing familial relationships (.ped format).

CAVaLRi expands this framework to account for large phenotype sets (generated manually or via NLP, Supplemental Figure 1), variant segregation in a pedigree, and the relative importance of diagnostic concepts (Figure 2, see Supplemental Materials for expanded mathematical description). These concepts are represented as separate LRs and capture phenotypic (LR_pheno_), genotypic (LR_geno_), and variant segregation (LR_seg_) information. The relative importance of each component LR is established empirically via a statistical learning procedure that optimizes the accuracy of diagnostic variant classification (Supplemental Figure 2). CAVaLRi departs from the traditional LR definition by introducing these LR weights. To restore this loss of interpretability, the scaled composite LR (CAVaLRi score) is calibrated to diagnostic and nondiagnostic score distributions to yield a new posterior probability. CAVaLRi ultimately outputs a prioritized gene list sorted by these posterior probabilities.

### 2.3. Phenotype Likelihood Calculation

#### 2.3.1. Generation of a Given Patient's Set of Phenotypes

The phenotypic abnormalities in a patient suspected of RGD can be represented computationally by a set of HPO terms. Trained clinicians curate these phenotype sets manually by reviewing a patient's clinical record and determining the HPO terms most relevant to a patient's condition. This subjective process typically prioritizes HPO terms with a high potential of being associated with a genetic diagnosis (i.e., high information content terms [29]). Alternatively, phenotype sets can be generated by NLP algorithms designed to extract HPO terms from plain-text clinical notes. Computationally derived phenotype sets tend to be larger and have more noise than manually curated sets [30]. However, some level of noise can be expected in both methods. Conceptually, a full phenotypic description of a patient with an RGD will include HPO terms attributable to the RGD, nongenetic etiologies, and possibly terms related to a second RGD. In practice, one does not know which group a given term belongs to. Identifying the optimal set of HPO terms to focus on for a given patient is further complicated by the fact that many RGDs are sparsely annotated in the HPO compared to the complete phenotypic spectrum of the condition [31, 32].

To overcome these limitations, rather than attempting to model whether an individual HPO term is attributable to a given RGD, we mimic the typical approach of clinical genomicists by iteratively considering subsets of phenotype terms to identify the subset that best supports a given candidate diagnosis. Consider a patient who has been referred to a clinical geneticist for assessment due to manifestations of intellectual disability and sensorineural hearing loss, amongst other clinical features. Genome sequencing has identified a potentially pathogenic variant in a gene associated with hearing loss. While not as compelling as a potentially pathogenic variant in a gene associated with both intellectual disability and sensorineural hearing loss, this variant should not be discounted just because the intellectual disability is not explainable by the candidate variant. This permits a dual diagnosis; our example patient could be diagnosed with both monogenic nonsyndromic hearing loss and a different diagnosis that accounts for intellectual disability. We represent this thought process computationally by creating patient phenotype subsets with the terms most consistent with a candidate genetic diagnosis. This requires a two-step process. First, patient phenotype terms from the HPO are ranked by relevance in genetic disease. Second, the highest-ranking phenotypes are incrementally subsetted when calculating the phenotype LR (LR_pheno_).

Two distinct approaches were utilized to rank phenotypes, depending on whether the terms were derived manually or computationally. For manually derived clinician-assigned phenotypes, HPO terms were ranked by assigning information content (IC) scores to each term using the IC formulation provided in Phrank [29]. This IC score is a function of HPO phenotype-gene annotations, where more specific terms with fewer gene annotations relative to their ancestors in the HPO graph receive higher scores. Computational phenotype sets were generated by the NLP algorithm, ClinPhen, which ranks each HPO term by the number of occurrences of the term within a patient's medical record. The developers of ClinPhen demonstrated that term occurrences outperformed IC when ranking diagnostic genes [23].

#### 2.3.2. Estimation of LR_pheno_

Once phenotype terms are sorted according to IC or clinical text occurrences, disease likelihoods are calculated in the context of the phenotype set for all OMIM disease entries. For each disease entry, the likelihood of the disease being present, given the observed phenotypic abnormalities, is calculated. Each phenotype element is estimated individually and combined to represent the LR_pheno_ for the candidate disease. Estimating the likelihood that a specific phenotype is indicative of a particular disease is challenging given the sparsity of disease-phenotype frequency annotations within the HPO (average of 15.5 annotated terms/diseases). This is especially true when considering the myriad of possible disease-phenotype combinations (>17,000 HPO terms in the phenotypic abnormality subontology, >8,100 OMIM diseases). Thoughtful propagation of the null fields in the phenotype-disease matrix can increase annotation coverage and accuracy in modeling.

The HPO is structured as a directed acyclic graph, where specific phenotypic abnormalities (child terms) are grouped under more general terms (parent terms). For example, “short stature” (HP:0004322) is a specific manifestation of the broader category “abnormality of body height” (HP:0000002). This parent-child relationship in the HPO is termed an “is a” relationship. We can capitalize on this hierarchical structure to address the sparsity issue, as it allows for the inheritance of frequency annotations from child to parent (making general categories inherit specifics). By extension, frequency annotations can be passed from child to parent terms (imparting general knowledge from more specific terms). For example, if “pituitary dwarfism” occurs in 40% of patients with a given disease, then “short stature” (its parent term) also occurs in at least 40% of those patients. In cases where a parent term has multiple annotated child terms, the maximum frequency amongst the child nodes is selected. To extend the previous example, assume another child term of “short stature,” “birth length less than 3rd percentile” is annotated with a disease frequency of 20%. The true disease frequency of “short stature” lies somewhere between 40% and 60%, depending on the coexpression of these terms in the disease population. Unfortunately, coexpression frequencies are not available under the current HPO annotations. Given this limitation, only the child node with the highest disease frequency is considered.

**Figure 3:**
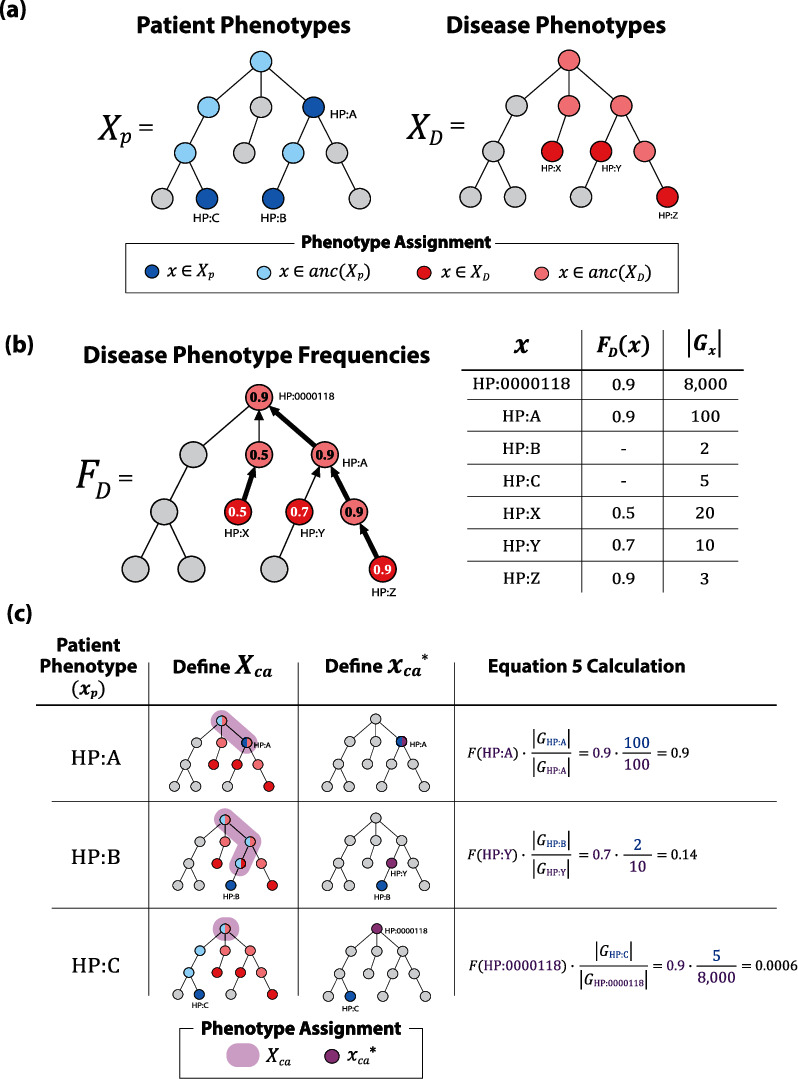
Calculating phenotype concordance between patient and disease phenotype sets. (a) Given a set of patient phenotypes (*X*_*p*_ = {HP : A, HP : B, HP : C}) and a set of disease phenotypes (*X*_*D*_ = {HP : X, HP : Y, HP : Z}), CAVaLRi calculates phenotype-disease concordance by iterating through each patient phenotype term *x*_*p*_ ∈ *X*_*p*_ and comparing to *X*_*D*_. First, the ancestral closure is determined for *x*_*p*_ ∈ *X*_*p*_ by selecting all nodes separating *x*_*p*_ from the root node (HP:0000118 in the case of the Human Phenotype Ontology, or HPO). Next, the ancestral closure of *X*_*D*_ (anc(*X*_*D*_)) is defined, which is a union of anc(*x*_*D*_) for *x*_*D*_ ∈ *X*_*D*_. (b) A disease-phenotype frequency lookup table (*F*_*D*_) is then populated by propagating annotated disease-phenotype frequencies up the HPO graph. When a node has more than one child node, the maximum disease-phenotype frequency amongst child nodes is assigned. Gene counts (|*G*_*x*_|) are indexed for all *x*_*p*_ ∈ *X*_*p*_ and *x*_*D*_ ∈ anc(*X*_*D*_). (c) Pr(*x*_*p*_|*D*) is calculated according to Supplemental Materials Equation 5 (*F*(*x*_*p*_)  when *x*_*p*_ ∈ anc(*X*_*D*_), *F*(*x*_*ca*_^∗^) · |*G*_*x*_*p*__|/|*G*_*x*_*ca*_^∗^_|, when *x*_*p*_ ∉ anc(*X*_*D*_)). For *x*_*p*_ ∈ *X*_*p*_, the set of common ancestors (*X*_*ca*_) is determined by intersecting anc(*x*_*p*_)  with anc(*X*_*D*_). The common ancestor term with the highest Pr(*x*_*p*_|*D*) (*x*_*ca*_^∗^) is identified and used to complete Equation 5 calculation.

In the context of a directed acyclic graph, the “ancestral closure” of a node (or term) refers to the set of nodes on any path drawn from the term of interest to the root node (Figure 3(a)). For the HPO, the root node is “phenotypic abnormality” (HP:0000118). So, if you select a term (like “pituitary dwarfism”), the ancestral closure would include every term (or nodes) you encounter as you trace back to “phenotypic abnormality”: “pituitary dwarfism” (is a), “short stature” (is a), “abnormality of body height” (is a), “growth delay” (is a), “growth abnormality” (is a), and “phenotypic abnormality.” By extension, we define the ancestral closure of a phenotype set to be all nodes (or terms) connecting a patient's phenotypes to the root node.

By propagating known disease-phenotype frequencies up the edges of the HPO graph, a frequency lookup table can be built that is keyed on HPO ID and stores the highest disease-phenotype frequencies across all descendent HPO terms (Figure 3(b)). This ancestrally closed phenotype representation contains more than four times as many values compared to the annotated terms alone (mean of 70.4 annotated terms/disease when ancestrally closed). The probability of observing the patient's phenotypes, given the presence of the candidate disease, is determined by querying the generated disease-frequency lookup table (Figure 3(c)). The probability of observing a patient's phenotypes, given the candidate disease is not present, is estimated by counting the number of OMIM diseases associated with each phenotype. These probabilities are used to calculate the LR_pheno_ of the patient's phenotype set. Lastly, as previously mentioned, CAVaLRi identifies the combination of phenotypes that most strongly support the diagnosis of each candidate disease (Supplemental Figure 1). The list of patient phenotypes, sorted by IC, is truncated to the *i* most informative terms over each iteration (*i* ranges from 1 to a configurable subset length maximum). Prior to iterating, an output vector is initialized to store the LR_pheno_ for each truncated subset. Starting with a subset of size one, the LR_pheno_ of the most informative term is stored in the first position of an output vector. Next, the LR_pheno_ of the two most informative terms are combined and stored in the second position of the output vector. This process continues until reaching a configured largest subset size, at which point a maximum is taken across all elements in the output vector. This value is returned as the LR_pheno_ for the patient's phenotype set for the candidate disease. This procedure identifies the subset of patient phenotypes that most support the candidate disease.

### 2.4. Genotype Likelihood Calculation

When prioritizing candidate variants, two procedures are required: (1) obtain a list of variants that could be diagnostic and (2) sort the list based on some calculated score. First, CAVaLRi preprocesses the provided variants to remove any that are unlikely to be considered pathogenic, such as those that are common to the population (i.e., variants with a high gnomAD frequency), artifactual (i.e., variants resulting from low-quality sequence coverage or alignment issues), or those that are unlikely to impact the translational process (i.e., those that occur in intronic regions). Second, following this filtering step, the likelihood of each genetic variant to cause disease (LR_geno_) is utilized to aid in sorting the candidate variant list.

#### 2.4.1. Variant Preprocessing

Before scoring variants for pathogenicity, provided variants are subjected to annotation and filtering to obtain a list of candidate variants. If artifactual regions are known, a file detailing the corresponding genomic coordinates can be provided. Any variants occurring in these regions will be removed. Configurable thresholds can also remove variants with quality or depth values lower than specified values. Validation is then applied to reference the proband sample column in the VCF and remove variants where the proband does not have an alternative allele. The resulting set of variants is normalized with bcftools [33] and annotated using the RefSeq, gnomAD, and ClinVar databases [34–36] via the ANNOVAR variant annotation tool [37]. Additional filtering is then applied to remove variants (1) occurring in frequencies greater than 1% in any gnomAD population, (2) located in a nonexonic region, or (3) synonymous functional effect. Variants occurring in canonical splice regions are rescued from the exonic filter, and variants with submitted pathogenic significance in ClinVar are retained regardless of the previously stated filtering logic. These filtered variants represent the case's candidate list of diagnostic variants and are subjected to LR_geno_ calculation.

#### 2.4.2. Estimation of LR_geno_

CAVaLRi relies on *in silico* pathogenicity prediction algorithms to identify variants that may be disease-causal. Algorithms were chosen that output the probability of pathogenicity for compatibility with the LR framework. Only variants with a higher probability of being pathogenic rather than benign (i.e., an *in silico* score greater than or equal to 0.5) are considered for evaluation. Any variant with a score less than 0.5 is heuristically omitted from further analysis. All variants are annotated as either exonic or intronic and scored accordingly.

Exonic variants resulting in reading frame shifts or alteration of start or stop codons are heuristically assigned a pathogenic probability of one. For nonsynonymous SNVs, CAVaLRi uses MetaRNN to predict pathogenicity [38]. For inframe indels, MutPred-Indel was utilized [39]. Of note, MutPred-Indel is computationally expensive and may be overridden in CAVaLRi to automatically assign nonframeshift indels a pathology score of 0.5. For intronic variants impacting a canonical splice site (within 2 base pairs of the splicing junction), SpliceAI is used to predict the pathogenic impact [40]. All other intronic variants are heuristically omitted. The CAVaLRi LR_geno_ is calculated with these pathogenicity predictions, variant population frequencies, and disease frequencies (see Mathematical Method Descriptions in Supplementary Materials). Additional heuristics are applied if one or more of the candidate variants in an associated gene are annotated as having pathogenic significance in ClinVar.

Of note, some genes are associated with multiple OMIM diseases. LR_geno_ is equivalent for all diseases associated with a gene, given that they share the same candidate variants. Thus, the LR_geno_ is calculated once for every gene containing a candidate pathogenic variant. If multiple variants are present within a single gene, the inheritance pattern for the associated candidate disease is considered. If the disease is dominant, the variant with the highest predicted pathogenicity is considered. If the disease is recessive, the top two scoring variants are averaged to obtain the predicted pathogenicity.

Candidate variants are further filtered at this stage as candidate variants transition to candidate genes. If the disease associated with the candidate gene is recessive, the gene must contain two predicted disease-causal alleles, whereas the dominant disease requires only one. If the number of alleles within the candidate gene is less than required, the gene is not scored and is heuristically omitted from further analysis. If a candidate gene is associated with both dominant and recessive diseases, a dominant mode of inheritance is assumed.

### 2.5. Segregation Likelihood Calculation

A core component of reaching a genetic diagnosis is determining whether detected variants segregate as expected, given the known MOI of a disease. Fortunately, parental samples are often available in pediatric ES/GS [19]. A “segregation” LR (LR_seg_) is heuristically defined as 10^−1^ in the case where variant inheritance does not match the annotated MOI (thus allowing for the possibility of incomplete penetrance, which is not systematically annotated in OMIM), 10^1^ if the variant inheritance does match the annotated MOI (i.e., two candidate variants in a recessive disease gene, inherited *in trans*), and 10^0^ if inheritance cannot be determined due to lack of parental data. If only one parent is present, partial inheritance evidence is modeled by taking the square root of the calculated LR_seg_ for the available parental data.

### 2.6. Optimization of Relative LR Importance

CAVaLRi introduces an optimization step to statistically learn the relative importance of phenotype, genotype, and segregation. Hyperparameters (exponential constants) are introduced to differentially scale each LR (the LR_pheno_ constant is fixed to 1) and effectively capture the relative importance of each modeled component. This optimization also accounts for the various approximations and heuristics applied to determine each component LR may be. Constant values (*c*_1_, *c*_2_) were optimized to maximize the area under the precision-recall curve (PR AUC, see Accuracy Metric Selection below). To assess the generalizability of the approach, a train-test split was performed (70% training, 30% test), stratifying over diagnostic status, self-reported race, and biological sex to reduce any potential biases in model training (Table 1 and Supplemental Figure 2(a)). The training data were further partitioned into five cross-validation folds before searching the two-dimensional (*c*_1_, *c*_2_) scalar space (Supplementary Figure 2(b)). A greedy search optimization approach was employed, starting from the origin of scalar space and stepping in the direction that maximizes the PR AUC (Supplementary Figure 2(c)). Each training partition's optimal (*c*_1_, *c*_2_) coordinates were averaged. Following the optimization procedure, scalars of 2.29 and 3.69 were assigned to LR_geno_ and LR_seg_, respectively.

### 2.7. Accuracy Metric Selection

CAVaLRi posterior probabilities were utilized to sort a list of candidate variants for each case and generate a corresponding precision-recall (PR) curve. PR curves demonstrate that classifier performance is maintained despite the stark class imbalance between the total number of diagnostic versus nondiagnostic variants. Furthermore, utilizing PR curves allows for performance assessment in a cohort containing both diagnostic and nondiagnostic cases. Mean diagnostic gene rank is also reported in cases with a known diagnosis. Mean rank was selected over median rank, as median rank maximizes to one for any algorithm ranking the diagnostic variant first at least 50% of the time, while mean rank allows one to distinguish between algorithms exceeding this level of accuracy.

### 2.8. Algorithmic Comparison

The PR AUC and mean diagnostic variant rank are considered to measure the performance of CAVaLRi against other leading variant prioritization algorithms. The initial comparison considers manually curated phenotypes from the clinical ES and DDD validation cohorts. A second comparison in the clinical ES cohort evaluates algorithmic performance given either manually curated or computationally generated phenotype sets. Comparators include the Exomiser prioritizers (hiPHIVE, PhenIX), LIRICAL, and XRare. These algorithms were cited in a recent review as highly accurate and open-source, allowing for reliable benchmarking [41].

## 3. Results

**Figure 4:**
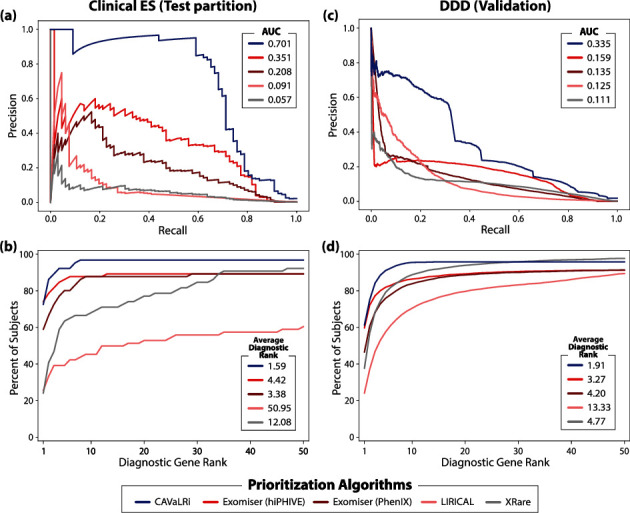
CAVaLRi demonstrates marked gains in diagnostic accuracy compared to existing methods in multiple patient cohorts. CAVaLRi was benchmarked against four leading diagnostic variant prioritization algorithms with clinician-curated phenotype sets (comparators include the hiPHIVE and PhenIX Exomiser prioritization, LIRICAL, and XRare). In the test partition for the clinical ES cohort, CAVaLRi displayed the highest accuracy as demonstrated by (a) higher PR AUC and (b) lower average diagnostic rank compared to all other algorithms. Similar relative performance was observed in the validation cohort (DDD) in terms of both (c) PR AUC and (d) average diagnostic rank.

When utilizing manually curated phenotypes in the clinical ES cohort, CAVaLRi output was more informative compared to all other algorithms in terms of both PR AUC (0.701) and average diagnostic gene rank (1.59; 72.7% of diagnostic genes were ranked in the first position) (Figures 4(a) and 4(b)). The Exomiser prioritizers were the next most accurate methods in terms of PR AUC (hiPHIVE: 0.351, PhenIX: 0.208) and average diagnostic gene rank (hiPHIVE: 4.42, 74.2% first position; PhenIX: 3.38, 59.1% first position). LIRICAL (PR AUC = 0.091, average diagnostic rank = 50.95, 25.8% first position) and XRare (PR AUC = 0.057, average diagnostic rank = 12.08, 24.2% first position) were less accurate than all other comparators. Of note, LIRICAL seems to run in global mode when TSV formatted output is requested. HTML formatted output was also generated to curb this behavior, but the diagnostic variant recall was inadequate. Diagnostic recall, or the percentage of diagnostic genes recovered after applying variant preprocessing (different for each comparator), was high across all algorithms in the clinical ES test partition (CAVaLRi: 97.0%, hiPHIVE: 90.9%, PhenIX: 90.9%, LIRICAL: 100%, and XRare: 97.0%). CAVaLRi did not recall two diagnostic genes due to one variant having a high population frequency and one variant having a low MetaRNN score. Differences in preprocessing resulted in differing numbers of candidate genes requiring review. Across the clinical ES cohort, CAVaLRi returned 2,925 candidate genes (averaging 14.8 per case), Exomiser returned 23,207 (117.8 per case, same preprocessing for hiPHIVE and PhenIX prioritizers), XRare returned 25,024 (127.0 per case), and LIRICAL returned 31,469 (159.7 per case).

In the DDD validation cohort, less accurate results were obtained for all algorithms (Figures 4(c) and 4(d)). The relative accuracy between algorithms was mostly consistent with the results observed in the clinical ES cohort both in terms of PR AUC (CAVaLRi: 0.335, hiPHIVE: 0.159, PhenIX: 0.135, LIRICAL: 0.125, and XRare: 0.111) and average diagnostic rank (CAVaLRi: 1.91, hiPHIVE: 3.27, PhenIX: 4.20, LIRICAL: 13.33, and XRare: 4.77). CAVaLRi placed the diagnostic gene in the first position more than all algorithms, at 61.4% (hiPHIVE: 59.8%, PhenIX: 46.6%, LIRICAL: 24.2%, and XRare: 37.7%). Recall of diagnostic genes was similar to that seen in the clinical ES test partition for all algorithms (CAVaLRi: 96.0%, hiPHIVE: 91.9%, PhenIX: 91.9%, LIRICAL: 98.6%, and XRare: 98.5%). Diagnostic genes that were not recalled by CAVaLRi include 95 with variants whose scores were below the indicated thresholds, 38 where RefSeq annotation indicated an intronic functional effect, and 13 where HPO annotations were not available for the disease associated with the diagnostic gene. CAVaLRi preprocessing returned the lowest number of candidate genes across the DDD cohort at 175,827 (averaging 13.7 per case). Exomiser returned 1,475,485 candidate genes (115.7 per case), XRare returned 791,638 (61.7 per case), and LIRICAL returned 1,034,646 (80.6 per case).

**Figure 5:**
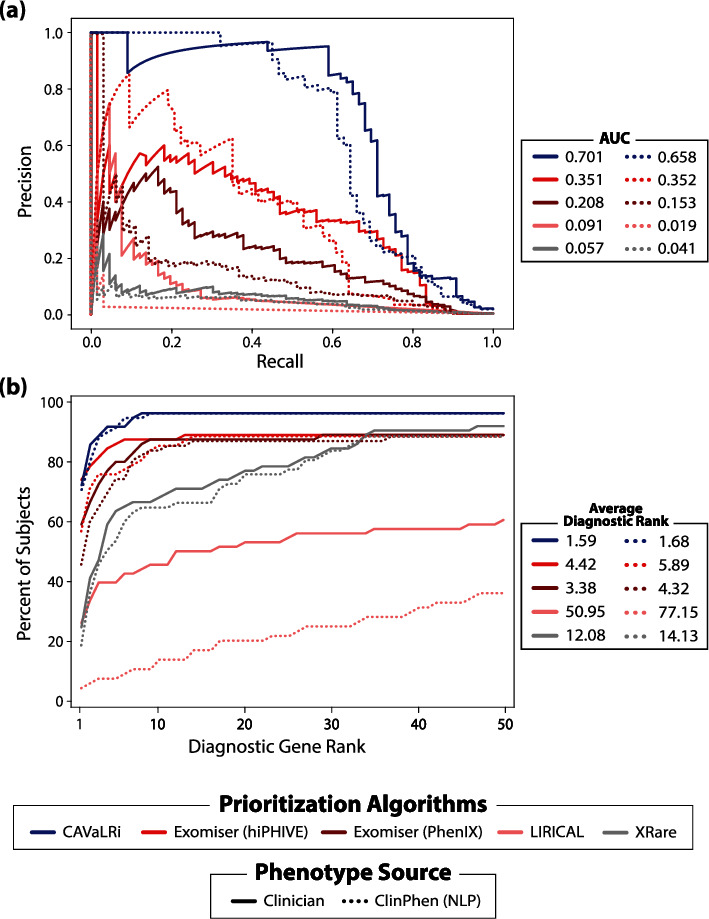
CAVaLRi remains equally performant regardless of the source of phenotype terms. The performance of all comparators was evaluated using sets of phenotype terms that were either curated by clinicians (“manual”; solid lines) or generated computationally through NLP processing of medical records (“ClinPhen”; dashed lines) from the test partition for the clinical ES cohort. Accuracy was measured again by (a) PR AUC and (b) top-N curves. CAVaLRi was slightly less accurate in terms of PR AUC and nearly identical in terms of average diagnostic rank when provided with computationally derived phenotype sets. By contrast, the other four algorithms evaluated, hiPHIVE, PhenIX, LIRICAL, and XRare, demonstrated a significant performance decrease when utilizing computationally generated phenotypes.

When computational phenotypes were substituted for manually curated phenotypes in the clinical ES test partition, CAVaLRi was the only algorithm to maintain high levels of accuracy when compared to performance with manually curated phenotypes (Figure 5). The accuracy of all algorithms generally decreased according to both PR AUC (reductions were CAVaLRi: 6%, hiPHIVE: 0%, PhenIX: 26%, LIRICAL: 79%, and XRare: 28%) and average diagnostic rank (reductions were CAVaLRi: 6%, hiPHIVE: 33%, PhenIX: 28%, LIRICAL: 51%, and XRare: 17%). Overall, CAVaLRi was markedly resilient to the use of potentially noisy computational phenotypes and continued to display more accurate results compared to all other algorithms according to both PR AUC (CAVaLRi: 0.658, hiPHIVE: 0.352, PhenIX: 0.153, LIRICAL: 0.019, and XRare: 0.041) (Figure 5(a)) and average diagnostic rank (CAVaLRi: 1.68, hiPHIVE: 5.89, PhenIX: 4.32, LIRICAL: 77.15, and XRare: 14.13) (Figure 5(b)). CAVaLRi placed the diagnostic gene in the first position more than all algorithms, at 71.0% (hiPHIVE: 56.5%, PhenIX: 45.2%, LIRICAL: 3.23%, and XRare: 17.7%). The order of comparator algorithm performance did not change between the two accuracy metrics, except for XRare outperforming LIRICAL in terms of PR AUC when provided with computationally-derived phenotypes.

The posterior probabilities appeared well-calibrated, with predicted diagnostic probabilities in the clinical ES test set closely aligning with observed diagnostic frequencies (Supplemental Figure 4(a, b)). Posterior probabilities in the DDD cohort were slightly higher than observed probabilities, particularly when the posterior probability was greater than 50%.

**Figure 6:**
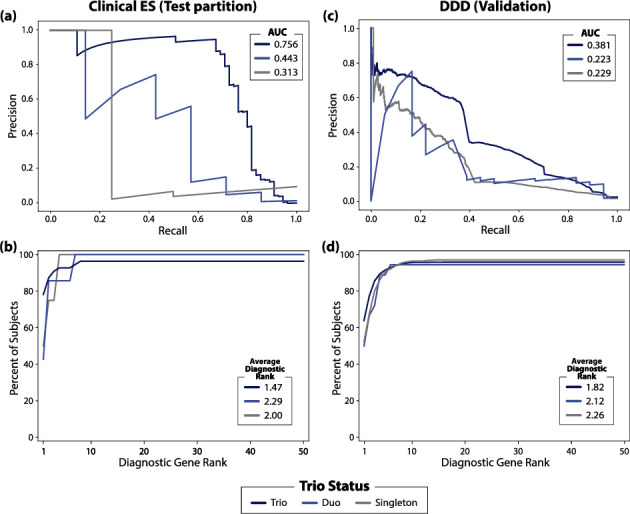
CAVaLRi's diagnostic prioritization accuracy is highly dependent on available parental data. Differences in prioritization accuracy were explored between cases where ES was available for both parents (trio), only one parent (duo), or neither parent (singleton). The availability of parental data improved CAVaLRi performance in the clinical ES test partition when parental data, with trio cases outperforming duo cases, and duo cases outperforming singleton cases, as assessed by (a) PR AUC and (b) top-N curves. The same pattern was observed in the DDD validation cohort as assessed by (c) PR AUC and (d) top-N curves.

Considerable differences in CAVaLRi performance were observed based on the availability of parental GS data. In the clinical ES test set, algorithmic performance on trios outperformed performance in duos and singletons in terms of both PR AUC (Figure 6(a), trios: 0.756, duos: 0.443, and singletons: 0.313) and average diagnostic gene rank (Figure 6(b), trios: 1.47, duos: 2.29, and singletons: 2.00). The same pattern was observed in the DDD cohort for PR AUC (Figure 6(c), trios: 0.381, duos: 0.223, and singletons: 0.229) and average diagnostic gene rank (Figure 6(d), trios: 1.82, duos: 2.12, and singletons: 2.26).

Of note, when running CAVaLRi, the *run_mutpredindel* flag was set to *False* after initial attempts to process the cohort data consumed exorbitant computational resources. This has been designated as the default value in the CAVaLRi settings.

## 4. Discussion

Of the open-source algorithms available for us to evaluate, CAVaLRi proved to be the most accurate algorithm in prioritizing diagnostic variants. Moreover, our approach provides an automated and scalable solution to meet the growing demand for ES/GS. CAVaLRi promotes scalability more than comparator algorithms by reducing the number of candidate genes requiring review by 87.4% in the clinical ES cohort and 77.8% in the DDD cohort compared to the second-best comparators (Exomiser in clinical ES, XRare in DDD). Additionally, in RGD cases where diagnostic variants are present, the burden of detailed manual review is significantly reduced considering that CAVaLRi demonstrated the lowest average diagnostic variant rank. CAVaLRi accounts for the segregation of variants more comprehensively than any published, open-source gene prioritization algorithm we are aware of. This feature is helpful in cases of incomplete penetrance, where robust phenotype matching and implied biological significance can outweigh an incompatible inheritance pattern. The CAVaLRi LR_pheno_ and LR_geno_ outperformed their equivalents in the LIRICAL, indicating a more informative representation of both phenotypic and genotypic data in the CAVaLRi framework (Supplemental Figure 5). The CAVaLRi LR_geno_ was more informative in binary classification compared to LR_pheno_, while the opposite trend was observed in LIRICAL.

The significant gains in accuracy were primarily attributable to the hyperparameter optimization procedure, where each LR was scaled according to the learned relative importance (Supplemental Figure 6(a, b)). The relatively high scalar values assigned to the LR_geno_ and LR_seg_ are concordant with the hypothesis that the true phenotypic spectrum of many RGDs is, at best, poorly understood. Instead, disease-phenotype annotations often reflect the distribution of phenotypes amongst those who are severely affected and were likely initially identified and described as the “classic” forms of a particular RGD. Future efforts to identify phenotypes that fall outside of these canonical features, for example, through reverse phenotyping approaches [42], would likely increase the utility of the LR_pheno_. By extension, this implies that refitting hyperparameters will be necessary as curated biomedical knowledge evolves.

Sorting and filtering phenotypes by IC allowed for extracting phenotype signals from otherwise noisy computationally generated phenotypes. The phenotype filtering extension of CAVaLRi hedges against phenotypic noise by prioritizing phenotypes associated with an RGD and only considering the subset of phenotypic terms that most closely align with candidate diagnoses. Computationally generated phenotype sets include significantly more HPO terms (mean = 151.4, SD = 102.6) compared to manually curated phenotype sets (mean = 30.1, SD = 13.2) (Supplemental Figure 7), requiring significant filtering to avoid performance degradation. By considering all phenotypes rather than an informative subset, comparator algorithms are vulnerable to losing important phenotype signals amongst genetically irrelevant phenotype terms. This was evidenced by less accurate performance across all comparator algorithms when provided larger computational sets compared to smaller, manually curated phenotype sets. Of note, the initial description of LIRICAL simulated noisy manually curated phenotypes and demonstrated minimal degradation in performance. However, these phenotype sets were not representative of computational phenotype sets derived from clinical notes, as they were much smaller.

This result confirms that computational methods that perform phenotype extraction in a fraction of the time human experts can be used with the right prioritization algorithm to save time without substantially sacrificing accuracy. Here, we used one such open-source NLP algorithm, ClinPhen, to convert plain-text clinical notes into a prioritized list of patient phenotypes with high accuracy and speed [23]. CAVaLRi's accuracy fell 6% with computational versus manually curated phenotypes in terms of both PR AUC and average diagnostic rank. However, the other algorithms demonstrated marked reductions in performance, ranging from 17% to 51% reductions in average diagnostic rank. Even with this 6% performance reduction, CAVaLRi was more accurate with computational phenotypes than all comparators using manually curated phenotypes. Considering that manual phenotyping is time-consuming, this result demonstrates that CAVaLRi combined with clinical NLP algorithms could further support scaling the availability of ES/GS.

When machine learning techniques are involved in optimizing algorithmic performance, there is always a concern for overfitting the model to the training data or sampled cohort. By validating the accuracy of CAVaLRi in a large external validation cohort, we provide strong evidence of generalizability outside of our institution. The DDD cohort is unique in that 72% of cases without CNV findings are undiagnosed. At the time of writing, no published germline variant prioritization algorithms are trained or validated on data that includes patients suspected of RGD with nondiagnostic ES/GS. CAVaLRi addresses this significant gap in the literature, as enriched training sets of true positive cases are not representative of cases in the clinical genetics setting, where as many as two-thirds of ES/GS are nondiagnostic [17–21]. Our training cohort has not been enriched other than to include subjects suspected of RGD for which clinical ES was ordered. This likely contributed to the robust performance of CAVaLRi in the DDD external validation cohort. CAVaLRi is optimized according to PR curves that are plotted on a per-gene basis. Approaching a case as a collection of candidate diagnostic genes allows clinical genomicists to quickly determine if a case is likely to yield a diagnosis. Optimizing on average diagnostic rank would not yield this capacity, as negative predictive value is not considered.

Despite the widespread clinical adoption of ES/GS testing, most cases of suspected RGD referred for ES/GS do not receive a diagnosis. Interval reanalysis has been reported to yield a diagnosis in 5-20% of reanalyzed patients depending on the time between initial analysis and reanalysis [10, 43–46]. In an undiagnosed cohort, an unprioritized reanalysis would currently call for the review of hundreds of variants. This would require resources that no center possesses. CAVaLRi offers an open-source method to prioritize candidate genes in reanalysis, limiting the effort required to identify new genetic diagnoses. With this functionality in mind, CAVaLRi can accept a cohort of multiple patients as input and return summary files that indicate the most likely diagnostic genes across all provided patients, as well as the variants that led to the gene being prioritized.

While the observed gains in accuracy are promising, certain limitations exist. CAVaLRi does not currently consider CNVs or SVs, which are also known to contribute to RGD etiology. As SV callers and corresponding pathogenicity predictors mature, the functionality to consider SVs will be incorporated into the CAVaLRi framework. While CAVaLRi recalled diagnostic variants 96-97% of the time, a perfect recall was not achieved. CAVaLRi failed to recall variants not associated with a disease, heterozygous variants that caused recessive RGDs, and variants that failed to reach configured pathogenicity thresholds. While pathogenicity thresholds can be lowered to achieve higher diagnostic variant recall, CAVaLRi will fail to return variants that lack OMIM disease annotations or do not agree with the corresponding disease's annotated MOI. In addition to recall limitations, CAVaLRi deviates from the traditional LR framework, as LRs are scaled by hyperparameters. To combat the potential loss of interpretability, we compute a posterior probability of diagnosis that relies on fitted probability distributions of diagnostic and nondiagnostic variants in the clinical ES training cohort. While CAVaLRi posterior probabilities closely aligned with observed diagnostic rates in the clinical ES test cohort, probabilities were consistently higher than observed in the DDD cohort at high probability values. As such, we would recommend that posterior probabilities be calibrated to a local cohort before clinical implementation. When defining heuristics, we assume that background phenotype frequencies are equivalent to the average annotated frequencies across all OMIM diseases. The validity of this assumption likely varies depending on the clinical setting. If CAVaLRi is implemented in a care setting where fewer patients are likely to yield a diagnosis or certain diagnoses are more frequent than others, the prior diagnostic probability should be adjusted accordingly.

## 5. Conclusion

As scalable solutions become necessary for augmented variant interpretation, CAVaLRi may reduce the number of genetic variants requiring detailed, manual review. Additionally, the capacity of CAVaLRi to distill signals from computed phenotypes minimizes the need for manual phenotype curation. Overall, the diagnostic variant prioritization accuracy displayed across the test and validation cohorts supports using CAVaLRi as a computational tool for diagnostic gene prioritization in patients suspected of RGD.

## Figures and Tables

**Table 1:** Demographic distribution of reported cases.

	Clinical ES training	Clinical ES test	DDD
*n*	458	197	12832
Diagnostic variant count (%)			
0	315 (68.8)	137 (69.5)	9217 (71.8)
1	141 (30.8)	54 (27.4)	3551 (27.7)
2	2 (0.4)	6 (3.1)	63 (0.5)
3	—	—	1 (<0.1)
Diagnostic = true (%)	143 (31.2)	60 (30.5)	3615 (28.2)
Trio status (%)			
Singleton	31 (6.8)	18 (9.1)	3297 (25.7)
Duo	108 (23.6)	27 (13.7)	82 (0.6)
Trio	319 (69.6)	152 (77.2)	9453 (73.7)
Years from sign out (mean (SD))	4.52 (1.05)	4.41 (1.02)	—
Sign-out age (mean (SD))	7.66 (7.00)	6.63 (6.15)	—
Clinical notes = available (%)	400 (87.3)	186 (94.4)	—
Sex = M (%)	257 (56.1)	111 (56.3)	7843 (61.1)
Race (%)			
Asian	15 (3.3)	8 (4.1)	—
Black or African American	28 (6.1)	15 (7.6)	—
Multiple races	21 (4.6)	8 (4.1)	—
Unknown	33 (7.2)	13 (6.6)	—
White	361 (78.8)	153 (77.6)	—
Ethnicity (%)			
Hispanic or Latino	16 (3.5)	10 (5.1)	—
Not Hispanic or Latino	420 (91.7)	181 (91.9)	—
Unknown	22 (4.8)	8 (4.0)	—

The clinical ES training and test partitions were stratified by diagnostic variant presence and race. DDD demographic distribution is also provided where applicable, and not all demographic data were provided in the EGAS00001000775 download.

## Data Availability

CAVaLRi is published under an Open Source Initiative approved 3-Clause BSD License to ensure that any interested academic institution can perform optimization with their own cohorts and implement their own version of the algorithm in their respective diagnostic workflows. Code for the CAVaLRi algorithm is available at our GitHub repository (https://github.com/nch-igm/CAVaLRi). Due to limitations in informed consent, clinical notes, molecular data, and diagnostic data are not made publically available for subjects enrolled in NCH's Clinical ES cohort. DDD data, including sequencing, phenotype, and relevant metadata, are available via the European Genome-Phenome Archive (EGA) (https://ega-archive.org/studies/EGAS00001000775) following Data Access Committee (DAC) approval.

## Abbreviations

ACMG: American College of Medical Genetics
AMP: Association for Molecular Pathology
AUC: Area under the curve
CAP: College of American Pathologists
CAVaLRi: Clinical Assessment of Variants by Likelihood Ratios
CLIA: Clinical Laboratory Improvement Amendments
Clinical ES: Clinical exome sequencing
DDD: Deciphering Developmental Disorders study
EGA: European Genome-Phenome Archive
ES: Exome sequencing (formerly whole exome sequencing (WES))
GS: Genome sequencing (formerly whole genome sequencing (WGS))
HPO: Human phenotype ontology
IC: Information content
IGM: Institute for Genomic Medicine
indel: Short insertion or deletion
IRB: Institutional Review Board
LR: Likelihood ratio
MOI: Mode of inheritance
NCH: Nationwide Children's Hospital
NLP: Natural language processing
OHRP: Office for Human Research Protections
OMIM: Online Mendelian Inheritance in Man
LR_geno_: Genotype likelihood ratio
LR_pheno_: Phenotype likelihood ratio
LR_seg_: Variant segregation (mode of inheritance) likelihood ratio
PR: Precision-recall
PR AUC: Area under the precision-recall curve
RGD: Rare genetic disease
ROC: Receiver operating characteristic
ROC AUC: Area under the receiver operating characteristic curve
SD: Standard deviation
SNV: Single nucleotide variant
VCF: Variant call format.

## Supplementary Materials

Supplementary Materials Supplementary data includes the following figures and tables. Supplemental Figure 1: three plots demonstrating the optimization of phenotype set length (a and b) and minimum individual phenotype score (c). Supplemental Figure 2: illustrations of the CAVaLRi statistical learning procedure (a and b) and a heat map visualizing optimal scalar selection (c). Supplemental Figure 3: histogram (a) and a line plot (b) showing the distribution of diagnostic and nondiagnostic genes and the procedure for fitting probability distributions. Supplemental Figure 4: line plot (a) and scatter plot (b) of observed and expected diagnostic variant frequencies demonstrating the accurate modeling of posterior probability. Supplemental Figure 5: precision-recall (a) and top-N curves (b) demonstrating increased gains in accuracy following optimization of the relative importance of likelihood ratios. Supplemental Figure 6: histogram demonstrating that sets of phenotype terms are substantially larger when using computational approaches than manual curation. Supplemental Table 1: table of diagnostic DDD cases excluded due to diagnostic variants occurring in nonsplicing, intronic regions. Supplemental Table 2: table of diagnostic DDD cases excluded due to incompatible modes of inheritance.

## References

[1] Almli L. M., Ely D. M., Ailes E. C. (2020). Infant mortality attributable to birth defects — United States, 2003–2017. *MMWR. Morbidity and Mortality Weekly Report*.

[2] Wright C. F., FitzPatrick D. R., Firth H. V. (2018). Paediatric genomics: diagnosing rare disease in children. *Nature Reviews Genetics*.

[3] Bavisetty S., Grody W. W., Yazdani S. (2013). Emergence of pediatric rare diseases: review of present policies and opportunities for improvement. *Rare Diseases*.

[4] Schofield D., Rynehart L., Shresthra R., White S. M., Stark Z. (2019). Long-term economic impacts of exome sequencing for suspected monogenic disorders: diagnosis, management, and reproductive outcomes. *Genetics in Medicine*.

[5] 1000 Genomes Project Consortium (2015). A global reference for human genetic variation. *Nature*.

[6] Frazer K. A., Murray S. S., Schork N. J., Topol E. J. (2009). Human genetic variation and its contribution to complex traits. *Nature Reviews Genetics*.

[7] Rego S., Dagan-Rosenfeld O., Zhou W. (2018). High-frequency actionable pathogenic exome variants in an average-risk cohort. *Molecular Case Studies*.

[8] Richards S., Aziz N., Bale S. (2015). Standards and guidelines for the interpretation of sequence variants: a joint consensus recommendation of the American College of Medical Genetics and Genomics and the Association for Molecular Pathology. *Genetics in Medicine*.

[9] Rehm H. L., Alaimo J. T., Aradhya S. (2023). The landscape of reported VUS in multi-gene panel and genomic testing: Time for a change. *Genetics in Medicine*.

[10] Birgmeier J., Haeussler M., Deisseroth C. A. (2020). AMELIE speeds Mendelian diagnosis by matching patient phenotype and genotype to primary literature. *Science Translational Medicine*.

[11] Amberger J. S., Bocchini C. A., Schiettecatte F., Scott A. F., Hamosh A. (2015). OMIM.org: Online Mendelian Inheritance in Man (OMIM®), an online catalog of human genes and genetic disorders. *Nucleic Acids Research*.

[12] Köhler S., Gargano M., Matentzoglu N. (2021). The Human Phenotype Ontology in 2021. *Nucleic Acids Research*.

[13] SoRelle J. A., Pascual J. M., Gotway G., Park J. Y. (2020). Assessment of interlaboratory variation in the interpretation of genomic test results in patients with epilepsy. *JAMA Network Open*.

[14] Lincoln S. E., Truty R., Lin C.-F. (2019). A rigorous interlaboratory examination of the need to confirm next-generation sequencing-detected variants with an orthogonal method in clinical genetic testing. *The Journal of Molecular Diagnostics*.

[15] Lincoln S. E., Hambuch T., Zook J. M. (2021). One in seven pathogenic variants can be challenging to detect by NGS: an analysis of 450,000 patients with implications for clinical sensitivity and genetic test implementation. *Genetics in Medicine*.

[16] Deignan J. L., Chung W. K., Kearney H. M. (2019). Points to consider in the reevaluation and reanalysis of genomic test results: a statement of the American College of Medical Genetics and Genomics (ACMG). *Genetics in Medicine*.

[17] Yang Y., Muzny D. M., Xia F. (2014). Molecular findings among patients referred for clinical whole-exome sequencing. *Journal of the American Medical Association*.

[18] Clark M. M., Stark Z., Farnaes L. (2018). Meta-analysis of the diagnostic and clinical utility of genome and exome sequencing and chromosomal microarray in children with suspected genetic diseases. *npj Genomic Medicine*.

[19] Tan T. Y., Lunke S., Chong B. (2019). A head-to-head evaluation of the diagnostic efficacy and costs of trio versus singleton exome sequencing analysis. *European Journal of Human Genetics*.

[20] Kumar R. D., Saba L. F., Streff H. (2023). Clinical genome sequencing: three years’ experience at a tertiary children’s hospital. *Genetics in Medicine*.

[21] McLean A., Tchan M., Devery S. (2023). Informing a value care model: lessons from an integrated adult neurogenomics clinic. *Internal Medicine Journal*.

[22] Yuan X., Wang J., Dai B. (2022). Evaluation of phenotype-driven gene prioritization methods for Mendelian diseases. *Briefings in Bioinformatics*.

[23] Deisseroth C. A., Birgmeier J., Bodle E. E. (2019). ClinPhen extracts and prioritizes patient phenotypes directly from medical records to expedite genetic disease diagnosis. *Genetics in Medicine*.

[24] Deciphering Developmental Disorders Study (2015). Large-scale discovery of novel genetic causes of developmental disorders. *Nature*.

[25] Wright C. F., Campbell P., Eberhardt R. Y. (2023). Genomic diagnosis of rare pediatric disease in the United Kingdom and Ireland. *The New England Journal of Medicine*.

[26] Kelly B. J., Fitch J. R., Hu Y. (2015). Churchill: an ultra-fast, deterministic, highly scalable and balanced parallelization strategy for the discovery of human genetic variation in clinical and population-scale genomics. *Genome Biology*.

[27] Robinson P. N., Ravanmehr V., Jacobsen J. O. B. (2020). Interpretable clinical genomics with a likelihood ratio paradigm. *American Journal of Human Genetics*.

[28] Smedley D., Jacobsen J. O. B., Jäger M. (2015). Next-generation diagnostics and disease-gene discovery with the Exomiser. *Nature Protocols*.

[29] Jagadeesh K. A., Birgmeier J., Guturu H. (2019). Phrank measures phenotype sets similarity to greatly improve Mendelian diagnostic disease prioritization. *Genetics in Medicine*.

[30] Robinson P. N., Haendel M. A. (2020). Ontologies, knowledge representation, and machine learning for translational research: recent contributions. *Yearbook of Medical Informatics*.

[31] Mishra R., Burke A., Gitman B. (2019). Data-driven method to enhance craniofacial and oral phenotype vocabularies. *The Journal of the American Dental Association*.

[32] Haimel M., Pazmandi J., Heredia R. J. (2022). Curation and expansion of Human Phenotype Ontology for defined groups of inborn errors of immunity. *The Journal of Allergy and Clinical Immunology*.

[33] Danecek P., Bonfield J. K., Liddle J. (2021). Twelve years of SAMtools and BCFtools. *Gigascience*.

[34] Landrum M. J., Lee J. M., Benson M. (2018). ClinVar: improving access to variant interpretations and supporting evidence. *Nucleic Acids Research*.

[35] Karczewski K. J., Francioli L. C., Tiao G. (2020). The mutational constraint spectrum quantified from variation in 141,456 humans. *Nature*.

[36] O'Leary N. A., Wright M. W., Brister J. R. (2016). Reference sequence (RefSeq) database at NCBI: current status, taxonomic expansion, and functional annotation. *Nucleic Acids Research*.

[37] Wang K., Li M., Hakonarson H. (2010). ANNOVAR: functional annotation of genetic variants from high-throughput sequencing data. *Nucleic Acids Research*.

[38] Li C., Zhi D., Wang K., Liu X. (2022). MetaRNN: differentiating rare pathogenic and rare benign missense SNVs and InDels using deep learning. *Genome Medicine*.

[39] Pagel K. A., Antaki D., Lian A. (2019). Pathogenicity and functional impact of non-frameshifting insertion/deletion variation in the human genome. *PLoS Computational Biology*.

[40] Jaganathan K., Panagiotopoulou S. K., McRae J. F. (2019). Predicting splicing from primary sequence with deep learning. *Cell*.

[41] Kelly C., Szabo A., Pontikos N. (2022). Phenotype-aware prioritisation of rare Mendelian disease variants. *Trends in Genetics*.

[42] Wilczewski C. M., Obasohan J., Paschall J. E. (2023). Genotype first: clinical genomics research through a reverse phenotyping approach. *American Journal of Human Genetics*.

[43] Ewans L. J., Schofield D., Shrestha R. (2018). Whole-exome sequencing reanalysis at 12 months boosts diagnosis and is cost-effective when applied early in Mendelian disorders. *Genetics in Medicine*.

[44] Xiao B., Qiu W., Ji X. (2018). Marked yield of re-evaluating phenotype and exome/target sequencing data in 33 individuals with intellectual disabilities. *American Journal of Medical Genetics. Part A*.

[45] Wright C. F., McRae J. F., Clayton S. (2018). Making new genetic diagnoses with old data: iterative reanalysis and reporting from genome-wide data in 1,133 families with developmental disorders. *Genetics in Medicine*.

[46] Bergant G., Maver A., Lovrecic L., Čuturilo G., Hodzic A., Peterlin B. (2018). Comprehensive use of extended exome analysis improves diagnostic yield in rare disease: a retrospective survey in 1,059 cases. *Genetics in Medicine*.

