# Supplementary material for: CAVaLRi: An Algorithm for Rapid Identification of Diagnostic Germline Variation
Source: Hum Mutat. 2024 Apr 29;2024:6411444. doi: 10.1155/2024/6411444 (PMC11918498; doi:10.1155/2024/6411444)
Supplement: Supplementary Materials — Supplementary data includes the following figures and tables. Supplemental Figure 1: three plots demonstrating the optimization of phenotype set length (a and b) and minimum individual phenotype score (c). Supplemental Figure 2: illustrations of the CAVaLRi statistical learning procedure (a and b) and a heat map visualizing optimal scalar selection (c). Supplemental Figure 3: histogram (a) and a line plot (b) showing the distribution of diagnostic and nondiagnostic genes and the procedure for fitting probability distributions. Supplemental Figure 4: line plot (a) and scatter plot (b) of observed and expected diagnostic variant frequencies demonstrating the accurate modeling of posterior probability. Supplemental Figure 5: precision-recall (a) and top-N curves (b) demonstrating increased gains in accuracy following optimization of the relative importance of likelihood ratios. Supplemental Figure 6: histogram demonstrating that sets of phenotype terms are substantially larger when using computational approaches than manual curation. Supplemental Table 1: table of diagnostic DDD cases excluded due to diagnostic variants occurring in nonsplicing, intronic regions. Supplemental Table 2: table of diagnostic DDD cases excluded due to incompatible modes of inheritance. [file 6411444.f1.pdf]

--- SUPPLEMENTAL MATERIALS ---

CAVaLRi: An Algorithm for Rapid Identification of Diagnostic Germline Variation

Robert J. Schuetz[1,2], Austin A. Antoniou[2], Grant E. Lammi[1], David M. Gordon[1], Harkness C. Kuck[1], Bimal P. Chaudhari[2,3,4,5†] and Peter White[1,2,3*]

[1] The Office of Data Sciences, The Abigail Wexner Research Institute, Nationwide Children's Hospital, Columbus, Ohio, USA

[2] The Steve and Cindy Rasmussen Institute for Genomic Medicine, The Abigail Wexner Research Institute, Nationwide Children's Hospital, Columbus, Ohio, USA

[3] Department of Pediatrics, The Ohio State University College of Medicine, Columbus, Ohio, USA

[4] Divisions of Neonatology, Genetics and Genomic Medicine, Nationwide Children's Hospital, Columbus, OH, USA.

[5] Center for Clinical and Translational Science, The Ohio State University and Nationwide Children's Hospital, Columbus, OH, USA.

[*] **Corresponding Author:** Peter White, Ph.D., Battelle Endowed Chair for Quantitative and Computational Medicine, The Steve and Cindy Rasmussen Institute for Genomic Medicine, Nationwide Children's Hospital, 575 Children's Crossroad, Columbus, OH 43215. USA. Email: peter.white@nationwidechildrens.org

[†] **Co-Corresponding Author:** Bimal P. Chaudhari, MD, MPH, The Steve and Cindy Rasmussen Institute for Genomic Medicine, Nationwide Children's Hospital, 575 Children's Crossroad, Columbus, OH 43215. USA. Email: bimal.chaudhari@nationwidechildrens.org

Keywords: clinical genomics, variant prioritization, variant interpretation, rare disease, genetics

Running title: Probabilistic ranking of genomic variation

Mathematical Description

Clinical Assessment of Variants by Likelihood Ratio (CAVaLRi) framework overview

LRs are advantageous, intuitive, and can be converted to posterior probabilities given some observed diagnostic evidence (E) and a prior probability (p). "Diagnostic evidence" in this context is a set of variant features identified by genomic sequencing, their segregation in a pedigree, and phenotypic information derived from a patient chart (manually or via NLP). Using a formulation of Bayes' theorem tailored for the LR context, these posterior probabilities are highly interpretable when determining if a given disease is present (Equation 1). $\Pr(D|E)$ is the posterior probability, i.e., the probability of disease D being present given diagnostic evidence E. p is the prior probability, which represents our initial belief or estimate of the likelihood of the disease before seeing any diagnostic evidence. LR_D is the LR for disease D, which quantifies how many times more likely a disease is to be present given E compared to its absence. This equation is used to update our initial belief (prior probability) based on new evidence (phenotypes and ES/GS results) to produce a revised belief (posterior probability).

$$\Pr(D \mid E) = \frac{p \cdot \mathrm{LR}_D}{(1-p)+(p \cdot \mathrm{LR}_D)} \quad (1)$$

Components of the clinical diagnosis workflow can be modeled by separate LRs representing the contribution of phenotypic (LR_{pheno}), genotypic (LR_{geno}), and segregation (LR_{seg}) information to posterior belief in a given diagnosis. The product of these individual LRs are equivalent to a single composite LR (LR_D) (Equation 2).

$$\mathrm{LR_D} = \mathrm{LR_{pheno}} \cdot \mathrm{LR_{geno}}^{c_1} \cdot \mathrm{LR_{seg}}^{c_2} \quad (2)$$

CAVaLRi departs from the traditional LR definition by introducing a statistical learning technique. This technique empirically corrects for misspecification of the

component LRs by optimizing the relative weights between LRs (c_1,c_2), resulting in enhanced performance and accuracy **(Figure 2)**. Of note, CAVaLRi ultimately prioritizes genes rather than variants or diseases. As such, the highest-scoring disease associated with a given gene G is chosen to represent the CAVaLRi gene score (Equation 3). In simple terms, the equation states that the highest LR among all diseases associated with gene G (D_G) determines the LR for a gene (LR_G). If a gene is linked to multiple diseases, and one of those diseases has strong diagnostic evidence (high LR), then the gene will inherit that strong evidence score. This approach effectively elevates the most compelling evidence from the disease-specific level to the broader gene-associated context.

$$\text{LR}_G = \max_{D \in D_G} (\text{LR}_D) \qquad (3)$$

Phenotype Likelihood Calculation

Estimation of LRpheno

The CAVaLRi LR_{pheno} for a given disease (D) and a given patient phenotype (x_p) ($\text{LR}_{\text{pheno}D}(x_p)$) is calculated by dividing the probability of observing phenotype x_p given that disease D is present ($\Pr(x_p|D)$) by the probability of observing phenotype x_p in an instance of any other RGD except D ($\Pr(x_p|\neg D)$). This can be rewritten as a quotient of conditional probabilities. To limit the capacity for relatively small values to penalize the overall phenotype score, the maximum is returned between the calculated quotient and a configurable minimum phenotype score (value of 1 determined by grid search optimization, Supplemental Figure 1(c); Equation 4):

$$\text{LR}_{\text{pheno}D}(x_p) = \max\left(\frac{\Pr(x_p|D)}{\Pr(x_p|\neg D)}, 1\right) \qquad (4)$$

A disease-phentoype frequency map for the candidate disease (F_D) must be generated before calculating $\Pr(x_p|D)$. If a term x has a frequency for disease D in HPO annotations, we set $F_D(x)$ to be equal to that frequency; however, if x has descendants with disease D frequency annotations, we set $F_D(x)$ to be the maximum of descendent frequencies. When a patient phenotype term x_p lies within the ancestral closure of any term associated with disease D (X_D), we define $\Pr(x_p|D)$ to be equal to the value stored in the propagated frequency map, $F_D(x)$. If x does not belong to the ancestral closure of X_D, the set of most recent common ancestors are determined between x_p and X_D, which we denote by X_{ca}. For each common ancestor term $x_{ca} \in X_{ca}$, a candidate value of $\Pr(x_p|D)$ is calculated by taking the product of the x_{ca} disease frequency, $F_D(x_{ca})$, and the ratio of the genes associated with x_p, (G_{x_p}, gene count $|G_{x_p}|$), versus the number of genes associated with x_{ca}, ($G_{x_{ca}}$, gene count $|G_{x_{ca}}|$). This penalty effectively drives candidate values of $\Pr(x_p|D)$ lower the farther x_{ca} is in the ontology. The maximum score amongst all candidate values is taken to estimate $\Pr(x_p|D)$. Of note, the x_{ca} that maximizes $\Pr(x_p|D)$ is almost always the closest to x_p in the HPO graph due to the relatively less penalizing value of $\frac{|G_{x_p}|}{|G_{x_{ca}}|}$ (smallest value of $|G_{x_{ca}}|$). This property is not guaranteed in the presence of multiple parentage, which is why $|G_{x_{ca}}|$ is calculated for all $x_{ca} \in anc(X_{ca})$. We denote the x_{ca} that maximizes $\Pr(x_p|D)$ as $x_{ca}{}^*$. (**Figure 3(c)**, Equation 5).

$$\Pr(x_p|D) = \begin{cases} F_D(x_p) & , x_p \in anc(X_D) \\ \max_{x_{ca} \in X_{ca}} F_D(x_{ca}) \cdot \frac{|G_{x_p}|}{|G_{x_{ca}}|} = F_D(x_{ca}{}^*) \cdot \frac{|G_{x_p}|}{|G_{x_{ca}{}^*}|} & , x_p \notin anc(X_D) \end{cases} \quad (5)$$

We estimate $\Pr(x_p|\neg D)$ using the HPO annotations table, specifically dividing the number of RGDs associated with $x_{ca}{}^*$ by the total number of diseases with phenotype-disease annotations. In essence, more specific terms will have lower frequencies, while more general terms will have higher frequencies due to associations with multiple diseases. For example, the Kayser-Fleischer ring (HP:0200032) is a grey-green or brownish-pigmented ring around the edge of the cornea. Currently, this term is only associated with a single disease, Wilson disease (OMIM: 277900, an RGD that prevents the body from removing extra copper). Given the uniqueness of the term-disease association and the fact that HP:0200032 $\in anc(X_{\text{OMIM: 277900}})$ ($x_{ca}{}^*$ = HP:0200032), the $\Pr(x_p|\neg D)$ for Kayser-Fleischer ring would be $\frac{1}{|\text{OMIM diseases}|}$. However, the parent term of Kayser-Fleischer ring is corneal opacity (HP:0007957), a term with 19 child terms associated with 329 diseases. This less specific term would have a $\Pr(x_p|\neg D)$ of $\frac{329}{|\text{OMIM diseases}|}$.

Multiple LRs for individual phenotypic abnormalities present in a patient can be multiplied together to compute an aggregate LR for a disease D and a set of patient phenotypes X_p. Assuming independence between phenotypic abnormalities $x_p \in X_p$, this aggregate LR can be calculated by taking the product of all $\text{LR}_{\text{pheno}D}(x_p)$ for $x_p \in X_p$ (Equation 6):

$$\text{LR}_{\text{pheno}D}(X_p) = \prod_{x_p \in X_p} \text{LR}_{\text{pheno}D}(x_p) \quad (6)$$

CAVaLRi introduces a novel, iterative procedure to detect phenotype-disease signal by incrementing the number of ordered patient phenotypes to consider (Supplemental Figure 1(a,b)). Initially, the top-ranked phenotype is assessed individually, followed by the product of scores for the top two phenotypes. This process continues, combining scores for the top-i ranked phenotypes

($\mathrm{LR}_D(X_{p(1,i)})$), until reaching a maximum of 19 phenotypes. (value of 19 determined by grid search optimization, Supplemental Figure 1(c)). For each subset, the value of $\mathrm{LR}_D(X_{p(1,i)})$ is stored in a vector. The maximum of these values is returned to represent the phenotypic evidence in support of a diagnosis of disease D (Equation 7):

$$\mathrm{LR}_{\mathrm{pheno}D} = \max_{1\le i\le 19} \mathrm{LR}_{\mathrm{pheno}D}(X_{p_{(1,i)}}) \qquad (7)$$

Genotype Likelihood Calculation

Once all variants are scored with functional region-specific pathogenicity *in silico* predictors, the CAVaLRi $\mathrm{LR}_{\mathrm{geno}}$ is calculated for all genes with possibly disease-causal variants. We define the CAVaLRi $\mathrm{LR}_{\mathrm{geno}}$ as follows (Equation 8):

$$\mathrm{LR}_{\mathrm{geno}} = \frac{\Pr(gt|D)}{\Pr(gt|\neg D)} \qquad (8)$$

By applying Bayes' Rule, $\Pr(gt|\, D)$ can be converted to conditional probabilities that are easier to calculate (Equation 9).

$$\frac{\Pr(gt|D)}{\Pr(gt|\neg D)} = \frac{\Pr(D|gt) * \Pr\,(gt)}{\Pr(gt|\neg D) * \Pr\,(D)} \qquad (9)$$

The probability of observing a genotype in the non-disease population ($\Pr(gt|\,\neg D)$) is roughly equivalent to observing a genotype in the general population, $\Pr(gt)$, given the relatively low incidence of any individual RGD. As such, these terms can be canceled in the final $\mathrm{LR}_{\mathrm{geno}}$ calculation (Equation 10).

$$\frac{\Pr(D|gt) * \Pr\,(gt)}{\Pr(gt|\neg D) * \Pr\,(D)} \approx \frac{\Pr(D|gt)}{\Pr(D)} \qquad (10)$$

By substituting the probability of being diagnosed with D given the patient's genotype ($\Pr(D|\, gt)$) with the obtained variant pathogenicity probabilities, the $\mathrm{LR}_{\mathrm{geno}}$ can be calculated by dividing $\Pr(D|\, gt)$ by prior probability of observing the disease ($\Pr(D)$). This

prior probability of observing the disease is estimated by sampling a uniform distribution of all possible OMIM diseases, or $\frac{1}{|\text{OMIM}|}$, assuming a described genetic disease is present.

The last nuance of LR_{geno} calculation relates to genetic variants annotated as having pathogenic or likely pathogenic significance, according to ClinVar. If a gene contains such a variant, the LR_{geno} is heuristically squared to model the emphasis placed on these variants during review and under ACMG guidelines [8].

Redefining posterior probability calculation

One obvious consequence of introducing hyperparameters is that the scaled LR no longer represents a true LR. To address this limitation, probability distributions were fitted to the CAVaLRi scores in diagnostic and non-diagnostic gene sets under the assumption of uniform prior probabilities for all diseases (Supplemental Figure 3(a,b)). Diagnostic odds can be calculated by comparing conditional probabilities of a given CAVaLRi gene score being sampled from either the diagnostic ($\Pr(\text{CAVaLRi score}|Diag)$ or non-diagnostic distribution ($\Pr(\text{CAVaLRi score}|\neg Diag)$ in context of prior frequencies ($\Pr(Diag)$ and $\Pr(\neg Diag)$, respectively) (Equation 11):

$$Diag_{\text{odds}} = \frac{\Pr(\text{CAVaLRi score}|Diag) * \Pr(Diag)}{\Pr(\text{CAVaLRi score}|\neg Diag) * \Pr(\neg Diag)} \quad (11)$$

Conditional probabilities are drawn from fitted probability distributions, while prior probabilities are estimated from all scored genes in the clinical ES training partition. From the diagnostic odds, the probability that a given variant is diagnostic can be calculated (Equation 12):

$$\Pr(Diag|\text{CAVaLRi score}) = \frac{1}{1 + Diag_{\text{odds}}} \quad (12)$$

This CAVaLRi definition of the posterior probability relies on probability distributions fitted to variant frequencies observed in the clinical ES training partition. Following variant preprocessing, CAVaLRi candidate variant lists are of similar length. This ensures that the ratio of diagnostic to non-diagnostic variants is comparable to those used to fit the probability distributions.

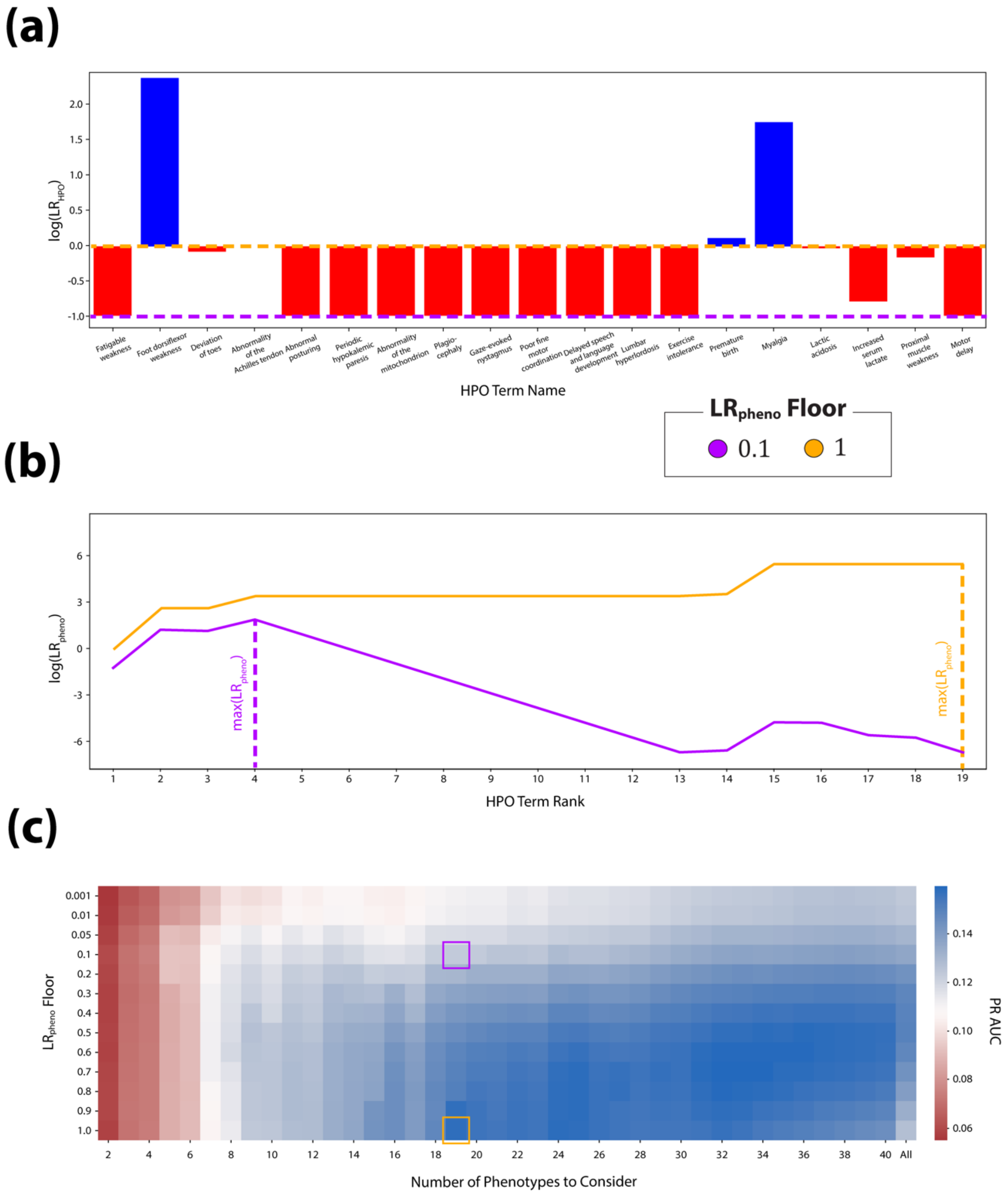

Supplemental Figure 1. Optimization of CAVaLRi LR_{pheno}. (**a**) Each phenotype term is first ordered based on information content, from left to right in this example with the most informative phenotyping being "Fatigable Weakness". All terms are then scored based on their relationship with the query disease's phenotype terms (Equation 4). The "floor" value effectively limits how much one phenotype can penalize the overall phenotype score. Illustrated are two phenotype score floor scenarios, 0.1 and 1. (**b**) The CAVaLRi LR_{pheno} of the patient's phenotype set is calculated by taking the maximum of the cumulative LR_{pheno} over

a range of phenotype set lengths. In this example, when individual term scores are floored at 0.1, the maximum LR_{pheno} occurs when the phenotype set contains 4 terms. When the floor value is increased to 1, the cumulative function is monotonically increasing, resulting in the maximum LR_{pheno} value occurring at the maximum set length value (19) (**c**) The selection of 19 as the maximum set length was determined empirically by greedy searching a two-dimensional hyperparameter space that includes maximum phenotype set length (x-axis) and the previously defined individual phenotype floor score (y-axis). The area under the precision-recall curve (PR AUC) of the maximum LR_{pheno} was chosen as the accuracy metric to optimize. Data from the training partition of the clinical ES cohort was utilized in calculating PR AUC. The greedy search was initiated at the origin of this hyperparameter space to favor smaller values. In the case of maximum phenotype set length, a smaller value would be favorable to accommodate cases with fewer phenotypes. A maximum phenotype set length of 19 and floor value of 1 were selected at the termination of the greedy search. Of note, PR AUC was lower when the floor value was set to 0.1 compared to 1, suggesting more accurate results when an individual term is not capable of penalizing the LR_{pheno} of the phenotype set.

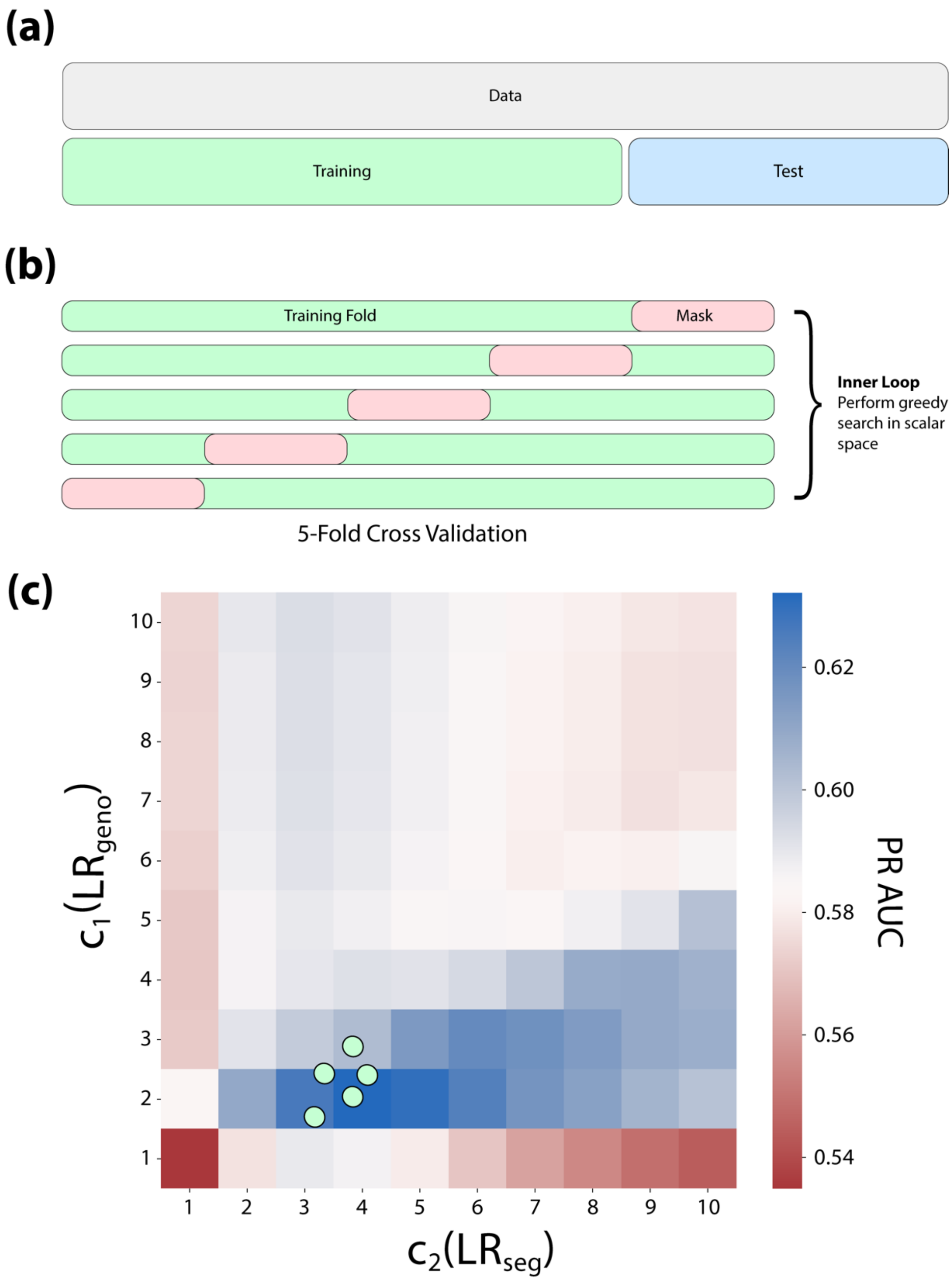

Supplemental Figure 2. CAVaLRi statistical learning procedure As opposed to available gene prioritization algorithms, CAVaLRi is trained to weight each component LR based on relative importance (Equation 2). (**a**) To ensure that CAVaLRi was naïve to a set of cases, the clinical ES cohort was split 70-30 into training and test partitions, respectively. (**b**) The training partition was further divided into 5 cross-folds to ensure that every training case

was masked during one iteration of hyperparameter tuning. (**C**) For each of the 5 iterations, unmasked training data was utilized in a greedy search procedure across two-dimensional scalar space to determine the relative importance of the LR_{geno} (c_1 in Equation 2) and LR_{seg} (c_2 in Equation 2). The greedy search was initialized at the origin. The area under the precision-recall curve (PR AUC) was selected as the optimized accuracy metric. Data from the training partition of the clinical ES cohort was utilized in calculating PR AUC. The arithmetic mean of the five optimization points (c_1, c_2) is the result of the statistical learning procedure. The returned mean optimization point values have been set as the default LR scalars in the CAVaLRi configuration settings ($c_1 = 2.29$, $c_2 = 3.69$).

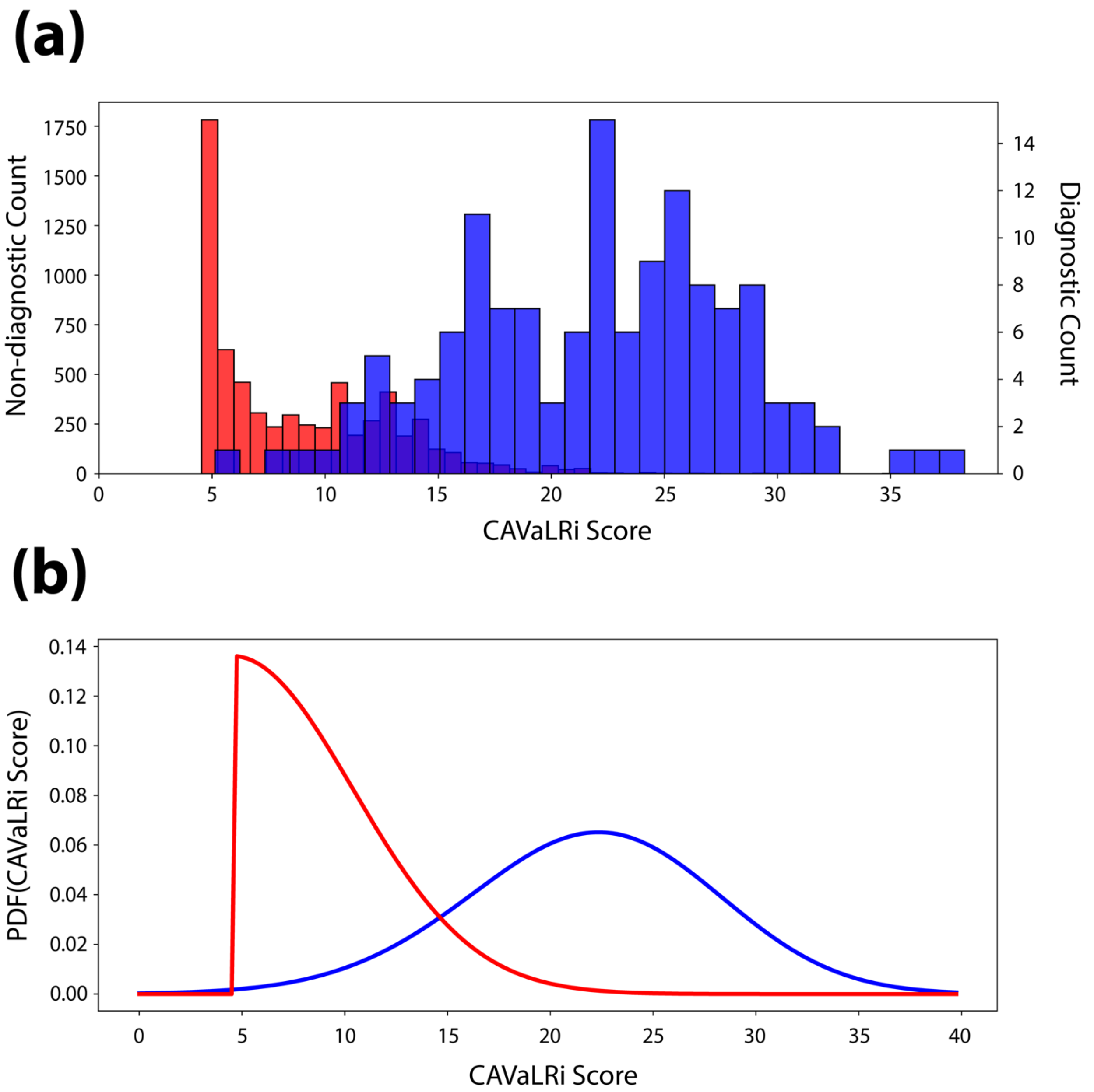

SUPPLEMENTAL FIGURE 3. Fitting probability distributions for diagnostic and non-diagnostic variants. (**a**) Histograms were generated from the CAVaLRi Score for all variants included in model training. Non-diagnostic variants are shown in red with counts indicated on the left y-axis; diagnostic variants are shown in blue with counts displayed on the right y-axis. (**b**) Skewed Gaussian distributions were fit individually for diagnostic and non-diagnostic variants. Associated probability density functions form the basis of the CAVaLRi posterior probability calculation (diagnostic parameters: α = -1.06 ζ = 26.25, ω = 7.59; non-diagnostic parameters: $\alpha = 1.38 * 10^7$, ζ = 4.53, ω = 5.86).

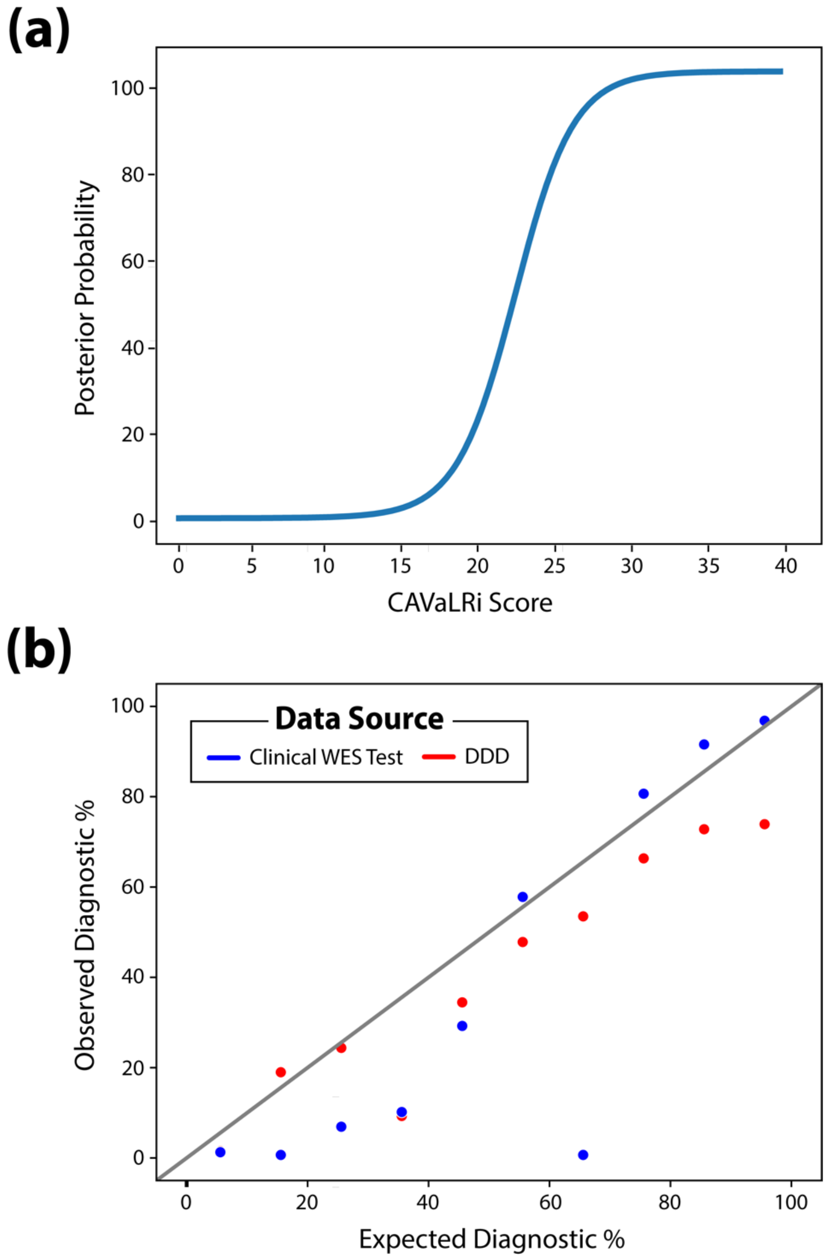

SUPPLEMENTAL FIGURE 4. Observed and expected diagnostic variant frequencies indicate accurate modeling of posterior probability. (**a**) Illustration of Equation 11 mapping CAVaLRi score to diagnostic posterior probability utilizing diagnostic and non-diagnostic Gaussian CAVaLRi score distributions. (**b**) Variants in the clinical ES test partition and DDD validation cohort were binned by predicted diagnostic probability (bin size 10). Average predicted values within these bins were used to calculate the expected diagnostic rate. The observed diagnostic rate within each bin was calculated by dividing the count of diagnostic variants by the total number of variants contained in each bin. The close alignment of expected and observed diagnostic rates supports the accuracy of the newly defined CAVaLRi posterior probability. Of note, CAVaLRi posterior probabilities were consistently higher than observed diagnostic ratios in the DDD cohort when diagnostic probability predictions were high. This may indicate that cohort calibration may be necessary in clinical settings.

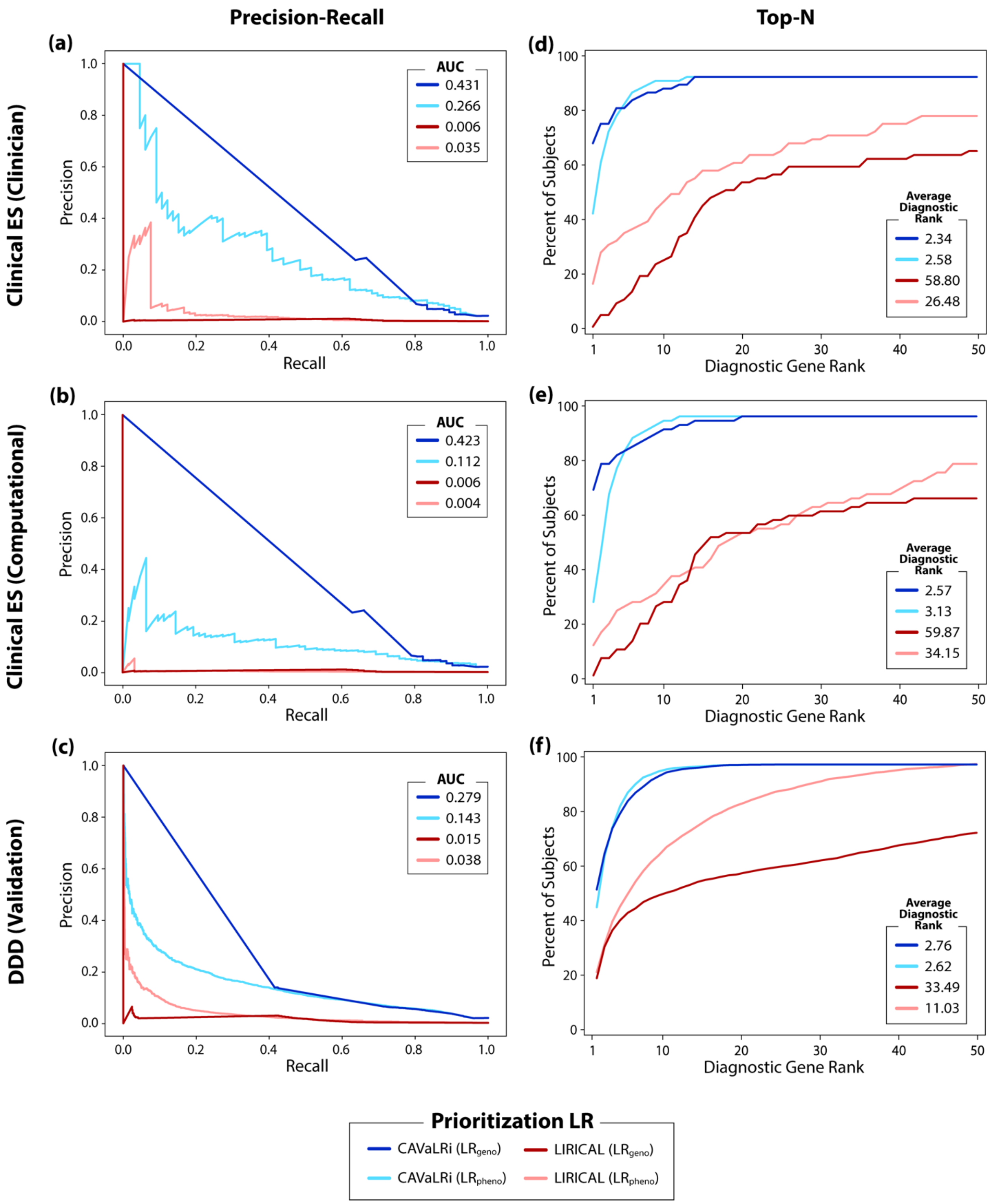

SUPPLEMENTAL FIGURE 5. Diagnostic variant classification accuracy by component likelihood ratio. CAVaLRi extends the likelihood ratio (**LR**) framework that was first described in LIRICAL. Precision-recall (**a-c**) and Top-N (**d-f**) curves are displayed to compare the diagnostic classification accuracy of the LRgeno and LRgeno for CAVaLRi and LIRICAL. Three cohorts are illustrated, the Clinical ES test partition with clinician-curated phenotypes

(Clinical ES (Clinician), **a-b**), the Clinical ES test partition with NLP-curated phenotypes (Clinical ES (Computational), **c-d**), and the DDD cohort (DDD (Validation), **e-f**). Generally, CAVaLRi component LRs outperformed their LIRICAL equivalents, the CAVaLRi LR_{geno} outperformed the LR_{pheno} (with the exception of the average diagnostic rank in the DDD cohort), and the LIRICAL LR_{pheno} outperformed the LR_{geno}. Of note, LIRICAL seems to run in global mode when TSV formatted output is requested. HTML formatted output was generated to obtain LR_{pheno} and LR_{geno} values.

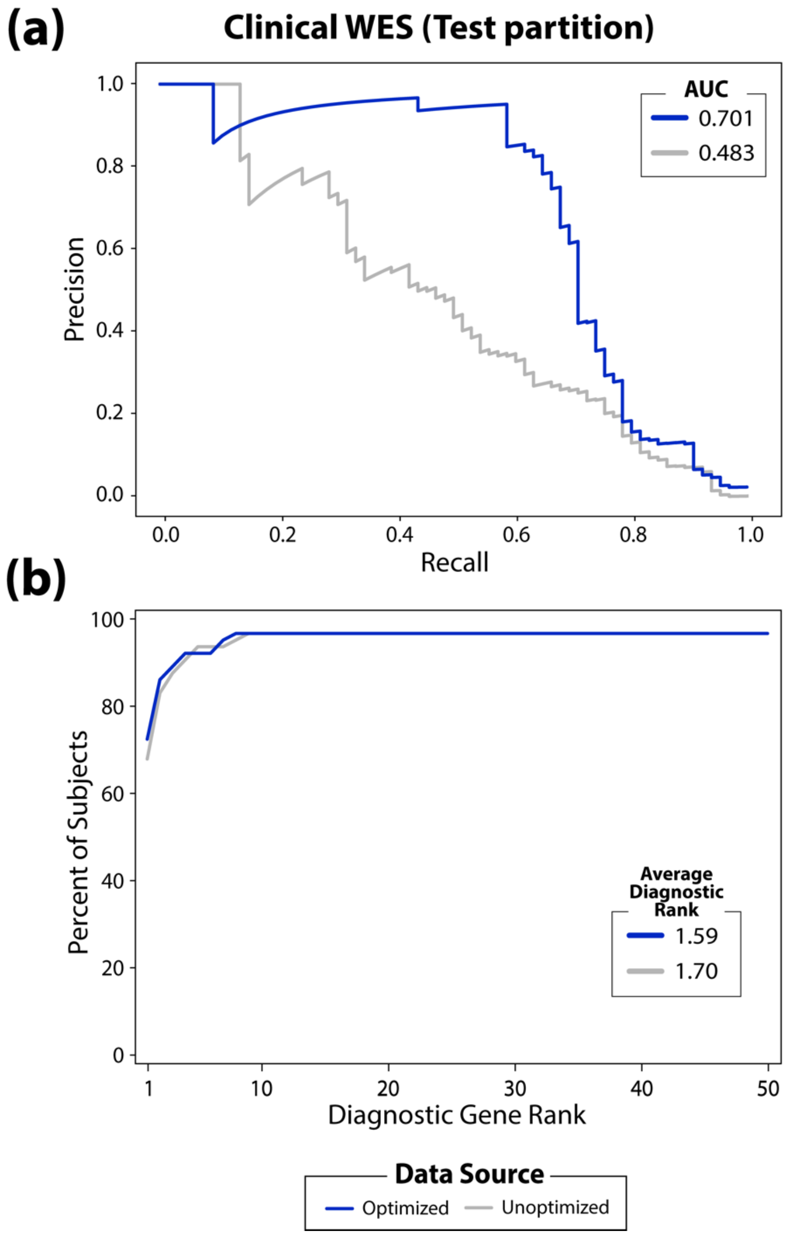

SUPPLEMENTAL FIGURE 6. Optimizing the relative importance of likelihood ratios leads to significant gains in accuracy. CAVaLRi achieves critical improvement in accuracy in the Clinical ES test partition after determining the relative importance of component likelihood ratios. CAVaLRi optimization yielded increases in both (**a**) precision-recall area under the curve (0.483 increased to 0.701) and (**b**) average diagnostic rank (1.70 decreased to 1.59)

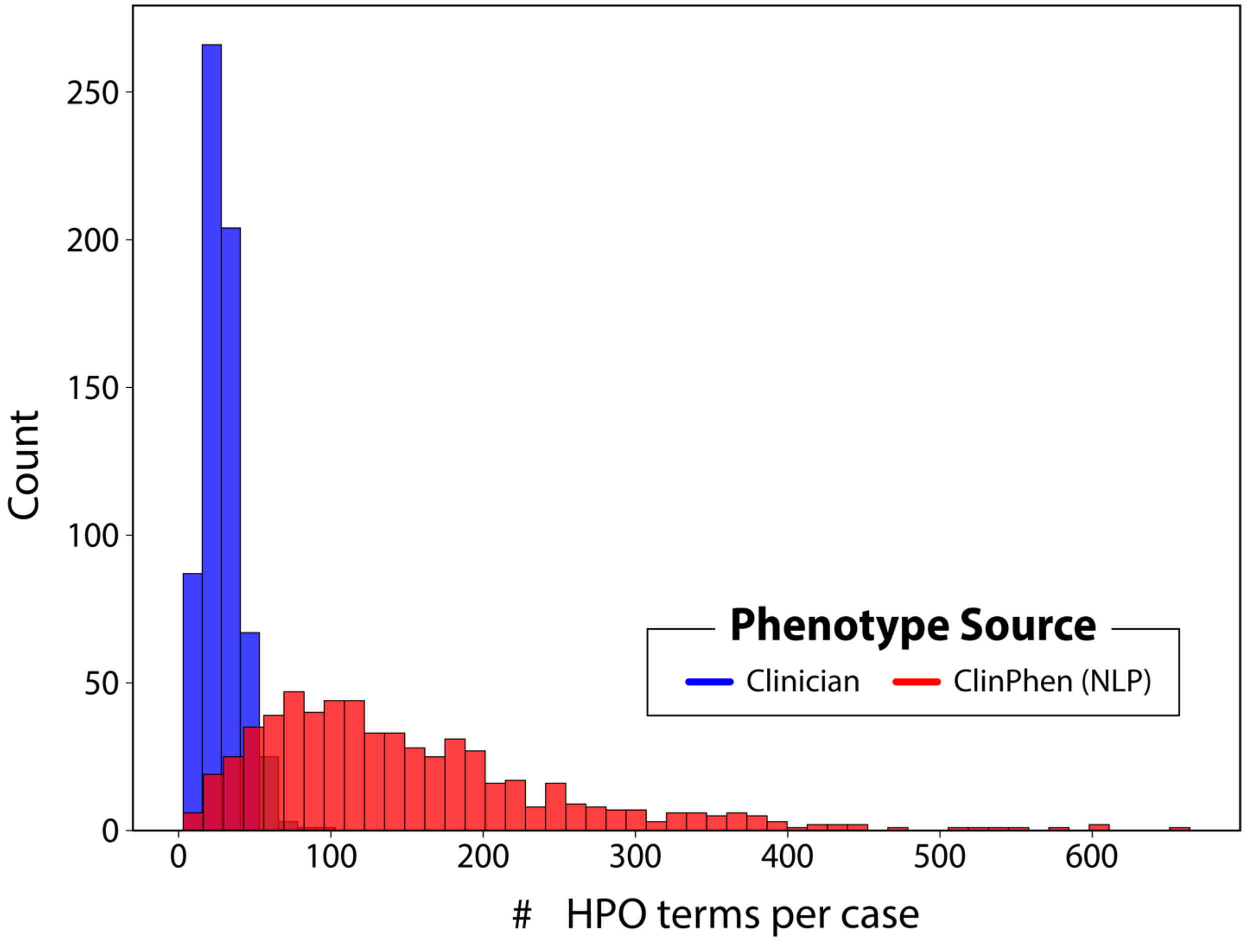

Supplemental Figure 7. Sets of phenotype terms are substantially larger when using computational approaches than manual curation. In practice, physicians manually review the clinical record to extract only the most genetically meaningful phenotype terms when ordering clinical ES testing. Natural language processing algorithms, namely ClinPhen, return far more terms on average than manually curated sets. Clinician curated set length (red): mean=30.1, SD=13.2; compared to computationally generated via ClinPhen (blue): mean=151.4, SD=102.6.

Case	Gene	Variant (GRCh38)	Reasoning
DDDP100135	SCN1A	2-165992435-A-T	Intronic for both selected and MANE transcripts
DDDP100153	NSD1	5-177238305-G-A	Synonymous for both selected and MANE transcripts
DDDP100209	KAT6B	10-75022006-G-A	Synonymous for both selected and MANE transcripts
DDDP100502	ITPR1	3-4815123-A-C	3' UTR for selected transcript, missense for MANE transcript
DDDP101064	KAT6B	10-75022006-G-A	Synonymous for both selected and MANE transcripts
DDDP101305	SETD5	3-9468525-T-A	Missense for selected transcript, intronic for MANE transcript
DDDP101570	SNRPB	20-2467306-C-G	Missense for selected transcript, intronic for MANE transcript
DDDP101628	NRXN1	2-49974149-T-G	Intronic for both selected and MANE transcripts
DDDP102250	TCF4	18-55228372-T-C	Intronic for both selected and MANE transcripts
DDDP103218	ITPR1	3-4814521-G-C	3' UTR for selected transcript, missense for MANE transcript
DDDP103895	MEF2C	5-88823891-C-T	5' UTR for both selected and MANE transcripts
DDDP104933	NKX2-1	14-36517958-G-C	3' UTR for selected transcript, missense for MANE transcript
DDDP106042	IFITM5	11-299504-G-A	5' UTR for both selected and MANE transcripts
DDDP106913	PORCN	X-48509783-G-A	5' UTR for selected transcript, splicing for MANE transcript
DDDP107539	IFITM5	11-299504-G-A	5' UTR for both selected and MANE transcripts
DDDP107924	HNRNPU	1-244862480-TGTGTCATCGAA-T	Intronic for selected transcript, frameshift for MANE transcript
DDDP109280	SMARCB1	22-23800933-T-G	Intronic for both selected and MANE transcripts
DDDP110855	CPLANE1	5-37226811-A-C	3' UTR for selected transcript, stopgain for MANE transcript
DDDP110961	ALG13	X-111685040-A-G	3' UTR for selected transcript, missense for MANE transcript
DDDP111138	NGLY1	3-25737407-G-A	Missense for selected transcript, synonymous for MANE transcript
DDDP111266	CASK	X-41555605-G-A	Intronic for selected transcript
DDDP111304	MEF2C	5-88823814-G-A	5' UTR for both selected and MANE transcripts
DDDP111509	CAMK2A	5-150228191-C-T	Intronic for selected transcript, splicing for MANE transcript
DDDP111580	ITPR1	3-4814497-G-A	3' UTR for selected transcript, missense for MANE transcript
DDDP112008	MEF2C	5-88823796-G-A	5' UTR for both selected and MANE transcripts
DDDP112091	HNRNPU	1-244862731-C-T	Intronic for selected transcript, splicing for MANE transcript
DDDP112101	GLI3	7-42023622-G-C	Intronic for both selected and MANE transcripts
DDDP112533	KMT2D	12-49027152-G-T	Synonymous for both selected and MANE transcripts
DDDP112668	WDR45	X-49076771-T-C	Missense for selected transcript, intronic for MANE transcript
DDDP112875	ITPR1	3-4815176-CAGA-C	3' UTR for selected transcript, inframe deletion for MANE transcript
DDDP114293	ST3GAL5	2-85848169-CT-C	Intronic for selected transcript, frameshift for MANE transcript
DDDP115427	STXBP1	9-127612378-C-G	5' UTR for both selected and MANE transcripts
DDDP115727	KAT6B	10-75022006-G-A	Synonymous for both selected and MANE transcripts
DDDP118412	ST3GAL5	2-85848203-CT-C	Intronic for selected transcript, frameshift for MANE transcript
DDDP118900	HNRNPU	1-244862714-TTC-T	Intronic for selected transcript, frameshift for MANE transcript
DDDP121248	ITPR1	3-4711828-T-C	3' UTR for selected transcript, missense for MANE transcript
DDDP121714	KCTD1	18-26548477-AGCGCTGGCGCTGCCGCCC-A	Intronic for selected transcript, inframe deletion for MANE transcript
DDDP122052	GRIA2	4-157361561-T-G	Missense for selected transcript, intronic for MANE transcript
DDDP122642	WT1	11-32391967-C-T	Intronic for selected transcript, splicing for MANE transcript
DDDP123526	DNM1	9-128226027-G-A	Splicing for selected transcript, intronic for MANE transcript
DDDP124153	WDR26	1-224431591-T-C	Intronic for both selected and MANE transcripts
DDDP125386	CREBBP	16-3769366-C-T	Intronic for both selected and MANE transcripts
DDDP125746	ZC4H2	X-64917770-G-GCT	Frameshift for selected transcript, 3' UTR for MANE transcript
DDDP125766	CPLANE1	5-37226580-A-C	3' UTR for selected transcript, missense for MANE transcript
DDDP125766	CPLANE1	5-37226811-A-C	3' UTR for selected transcript, stopgain for MANE transcript
DDDP125767	CPLANE1	5-37226580-A-C	3' UTR for selected transcript, missense for MANE transcript
DDDP125767	CPLANE1	5-37226811-A-C	3' UTR for selected transcript, stopgain for MANE transcript
DDDP127064	MEF2C	5-88823854-T-A	5' UTR for both selected and MANE transcripts
DDDP127760	SLC52A2	8-144360922-C-T	Missense for selected transcript, synonymous for MANE transcript
DDDP127855	MEF2C	5-88729356-A-C	Intronic for both selected and MANE transcripts
DDDP128881	ITPR1	3-4814521-G-A	3' UTR for selected transcript, missense for MANE transcript
DDDP128906	HUWE1	X-53625900-CGGGACT-C	Intronic for both selected and MANE transcripts
DDDP135483	HUWE1	X-53625871-G-GGGGCCA	Intronic for both selected and MANE transcripts
DDDP136425	KAT6B	10-75022006-G-A	Synonymous for both selected and MANE transcripts
DDDP137348	GFAP	17-44908075-G-A	3' UTR for selected transcript, missense for MANE transcript
DDDP137672	NIPBL	5-37022036-A-G	Intronic for both selected and MANE transcripts
DDDP138296	OFD1	X-13752714-C-A	Intronic for both selected and MANE transcripts
DDDP139049	ITPR1	3-4814521-G-C	3' UTR for selected transcript, missense for MANE transcript

SUPPLEMENTAL TABLE 1. Diagnostic DDD cases excluded due to diagnostic variants occurring in non-splicing, intronic regions. There were 56 diagnostic cases (58 variants) in the DDD cohort where the causal variant was in an intronic region not within 2 base pairs of an exonic splice region. These cases were omitted from the final analysis.

Case	Gene	Variant (GRCh38)	Zygosity	Biological Sex	Disease	MOI
DDDP102261	PHF6	X-134417289-C-T	Heterozygous	Female	OMIM:301900	XLR
DDDP104701	OGT	X-71559365-T-A	Heterozygous	Female	OMIM:300997	XLR
DDDP107584	CDT1	16-88807284-C-T	Heterozygous	Male	OMIM:613804	AR
DDDP109173	NRXN1	2-50922661-G-A	Heterozygous	Female	OMIM: 600565	AR
DDDP110885	ARHGEF9	X-63697176-A-T	Heterozygous	Female	OMIM:300607	XLR
DDDP111554	BRWD3	X-80693016-AC-A	Heterozygous	Female	OMIM: 300659	XLR
DDDP111896	ARHGEF9	X-63674082-T-TA	Heterozygous	Female	OMIM:300607	XLR
DDDP113450	FARS2	6-5613259-C-G	Heterozygous	Male	OMIM:614946	AR
DDDP115974	NRXN1	2-50472472-C-T	Heterozygous	Male	OMIM: 600565	AR
DDDP117224	SLC6A8	X-153694347-G-A	Heterozygous	Female	OMIM:300036	XLR
DDDP117298	OTC	X-38401372-G-C	Heterozygous	Female	OMIM:311250	XLR
DDDP121636	ARHGEF9	X-63706287-AG-A	Heterozygous	Female	OMIM:300607	XLR
DDDP123972	PHF6	X-134393557-T-A	Heterozygous	Female	OMIM:301900	XLR
DDDP126473	GRID2	4-93490747-T-C	Heterozygous	Male	OMIM:616204	AR
DDDP127185	KDM5B	1-202729961-C-A	Heterozygous	Female	OMIM:618109	AR
DDDP132170	CLCN5	X-50090678-C-T	Heterozygous	Female	OMIM: 300009	XLR
DDDP134103	NRXN1	2-51027934-A-AGG	Heterozygous	Male	OMIM: 600565	AR
DDDP134528	PHF6	X-134413956-A-G	Heterozygous	Female	OMIM:301900	XLR
DDDP135070	NRXN1	2-50538378-G-C	Heterozygous	Female	OMIM: 600565	AR

SUPPLEMENTAL TABLE 2. Diagnostic DDD cases excluded due to incompatible modes of inheritance. There were 19 instances where a diagnostic variant was called diagnostic despite contrasting the annotated mode of inheritance. These cases were omitted from the final analysis.
